# Knowledge transfer in lifelong machine learning: a systematic literature review

**DOI:** 10.1007/s10462-024-10853-9

**Published:** 2024-07-26

**Authors:** Pouya Khodaee, Herna L. Viktor, Wojtek Michalowski

**Affiliations:** 1 School of Electrical Engineering and Computer Science (EECS), University of Ottawa, 800 King Edward Avenue, Ottawa, ON K1N 6N5 Canada; 2 Telfer School of Management, University of Ottawa, 55 Laurier Avenue East, Ottawa, ON K1N 6N5 Canada

**Keywords:** Continual learning, Knowledge transfer, Lifelong machine learning, Transfer learning

## Abstract

**L**ifelong **M**achine **L**earning (LML) denotes a scenario involving multiple sequential tasks, each accompanied by its respective dataset, in order to solve specific learning problems. In this context, the focus of LML techniques is on utilizing already acquired knowledge to adapt to new tasks efficiently. Essentially, LML concerns about facing new tasks while exploiting the knowledge previously gathered from earlier tasks not only to help in adapting to new tasks but also to enrich the understanding of past ones. By understanding this concept, one can better grasp one of the major obstacles in LML, known as **K**nowledge **T**ransfer (KT). This systematic literature review aims to explore state-of-the-art KT techniques within LML and assess the evaluation metrics and commonly utilized datasets in this field, thereby keeping the LML research community updated with the latest developments. From an initial pool of 417 articles from four distinguished databases, 30 were deemed highly pertinent for the information extraction phase. The analysis recognizes four primary KT techniques: Replay, Regularization, Parameter Isolation, and Hybrid. This study delves into the characteristics of these techniques across both neural network (NN) and non-neural network (non-NN) frameworks, highlighting their distinct advantages that have captured researchers’ interest. It was found that the majority of the studies focused on supervised learning within an NN modelling framework, particularly employing Parameter Isolation and Hybrid for KT. The paper concludes by pinpointing research opportunities, including investigating non-NN models for Replay and exploring applications outside of computer vision (CV).

## Introduction

In traditional machine learning (ML), an algorithm is applied to a *single* dataset and then proceeds to construct a model. Once a model has been tested and deemed to represent the patterns in data, it may be deployed in production. As such, traditional ML algorithms learn in isolation, addressing one learning problem, so-called task (Liu and Cocea 2018). In contrast, Lifelong Machine Learning (LML) is an ML paradigm where there are *multiple sequential tasks*, each with its own corresponding data (Chen and Liu 2018). In the area of ML, researchers have drawn parallels between LML and Continual Learning (CL) due to their many shared characteristics. Consequently, these terms have often been used interchangeably in the literature (Mai et al 2022). However, one should acknowledge a debated point centred around the distinction between LML and CL. Chen and Liu (2018) state that the researchers who have focused on deep learning methods in LML, specifically deep neural networks, have used the CL term as a synonym for LML. Thus, LML is broader, where a branch of it that covers deep learning is known as CL. Hence, this study benefits from the term *lifelong machine learning* in its investigations due to its broader scope. This approach also guarantees the coverage of *continual learning* topics.

Humans have the ability to constantly acquire and pass on knowledge from one task/duty to another. This natural capacity is essential for developing skills related to our senses and movements and for storing and recalling long-term memories (Parisi et al 2019). In ML, the idea of utilizing prior knowledge to learn new tasks has also been explored. However, this approach involves the challenge of how to apply past knowledge to adjust to new tasks sequentially. This is where the concept of *knowledge transfer* (KT) comes into play and becomes essential. KT refers to the process of transferring knowledge/experience acquired from earlier experiences (tasks) and applying it to new situations, tasks or environments (Kudithipudi et al 2022). Essentially, KT in LML involves the model’s ability to adapt to new tasks by applying knowledge acquired from previous tasks (forward knowledge transfer), while simultaneously improving its comprehension of the tasks it has already learned (backward knowledge transfer).

The objective of this study is to conduct a systematic literature review (SLR) centred on state-of-the-art KT techniques in the LML domain. It also aims to identify the most often used evaluation metrics and benchmark datasets used by the LML community. Furthermore, the review intends to shed light on promising directions for future research in KT for LML, considering existing challenges. In this regard, the following research questions (RQs) are formulated:

- **RQ1:** What are the current and state-of-the-art techniques for KT in LML?
- **RQ2:** What evaluation metrics and benchmark datasets are utilized most frequently in studies focusing on KT in LML?

Based on the terms used in shaping this study’s search queries for addressing the aforementioned questions, 417 publications from four separate databases initially were retrieved and out of that 30 studies were identified as relevant to the RQs. It is worth noting that this study will consider the domains of Supervised, Unsupervised, and Reinforcement Learning.

While various studies have explored different aspects of LML, to the best of the authors’ knowledge, none have specifically concentrated on KT within the area of LML. The biological aspect of LML and its application in the domain of Neural Networks was explored by Parisi et al (2019), while Lesort et al (2020) delved deeper into dynamic environments of LML within the robotics field. The study by Pfülb and Gepperth (2019) focused on Catastrophic Forgetting, where learning new information can lead to the loss of previously acquired knowledge, proposing hyper-parameter tuning as a mitigation strategy. Mai et al (2022) reviewed online continual learning, particularly in image classification, addressing its current methods and challenges. Recently, Faber et al (2023) researched LML in anomaly detection. The study conducted by New et al (2022) primarily concentrated on LML metrics, but it did not specifically investigate metrics from the KT perspective. Despite these reviews, there remains a gap in research on KT techniques in LML. With the rapid evolution of methods and models in LML, the authors aim to keep researchers updated on the latest developments and lay the foundation for future advances in the field.

This paper is organized as follows. Section 2 provides an overview of LML and KT Techniques. Section 3 discusses the literature review methodology in detail. Sections 4 and 5 present the extracted data and the results obtained from the selected papers. Subsequently, Sect. 6 provides an analysis by the authors. This is followed by a discussion on the limitation in Sect. 7 and finally, the Conclusion in Sect. 8.

## Overview of LML and KT techniques

Following the introduction of LML and its key features, this section offers an overview of the techniques commonly employed for KT.

### Lifelong machine learning (LML)

**Fig. 1:**
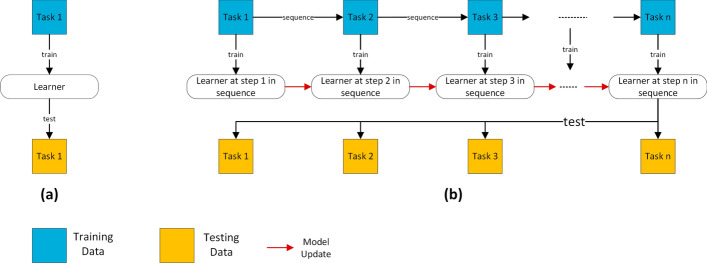
As depicted in **a** traditional ML systems are limited to a single task in which the learner is trained and tested on that task. In contrast, **b** illustrates the capabilities of LML, where the system can receive a sequence of tasks with their respective dataset and share the knowledge of the learner to learn new incoming tasks effectively. The learner is tested on the new and previous tasks. It is expected to keep the performance (i.e., evaluation metrics) on previous tasks, too, while learning and showing stable performance on new tasks

**Fig. 2:**
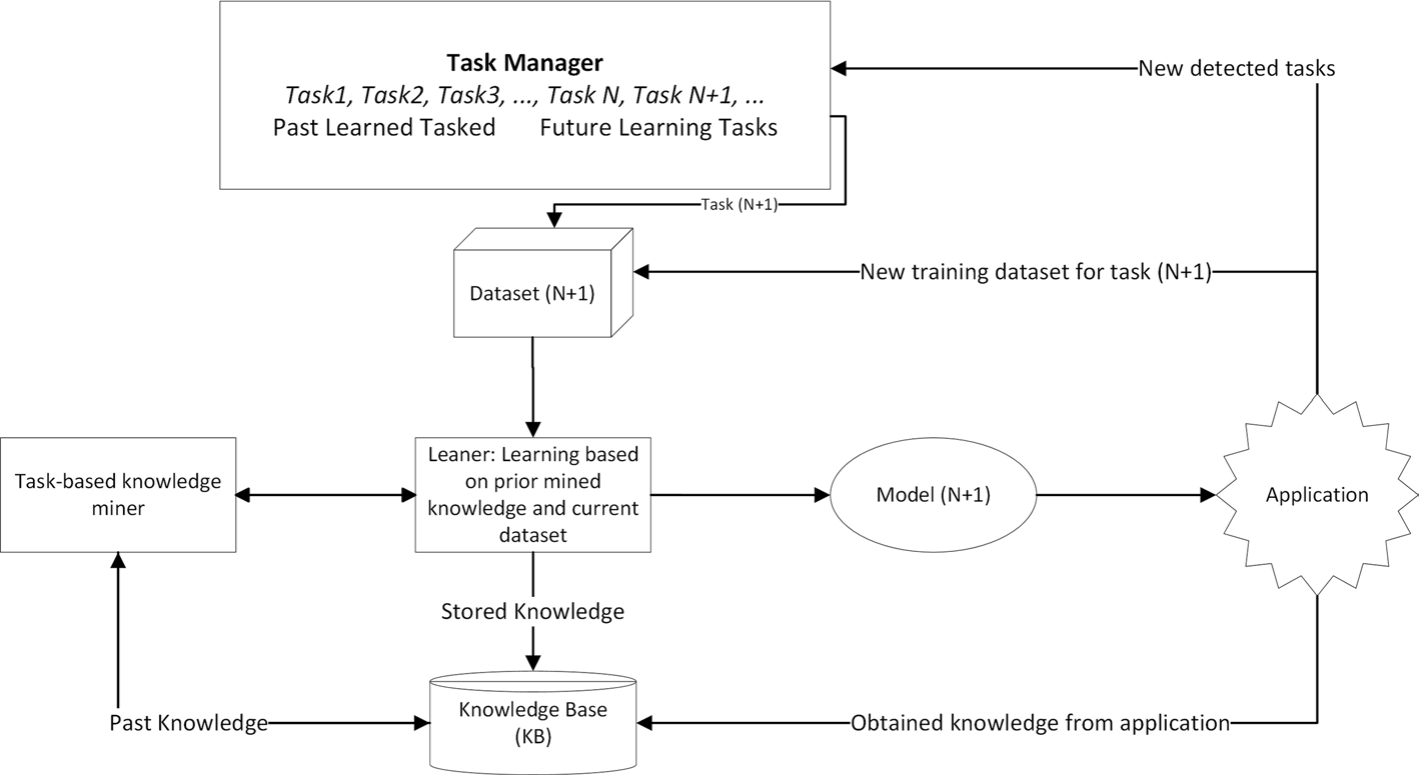
The system architecture of LML. The Knowledge Base (KB) serves as a repository for the acquired knowledge, including samples, pseudo-samples, model parameters, and other relevant information that will be discussed in this review

LML is an adaptive ML method designed to support continuous learning from datasets that arrive sequentially, where each dataset is associated with distinct task. Notably, LML methods operate without the need for a predetermined limit on the number of tasks to be learned (Parisi et al 2019). Within this framework, a *task* denotes a distinct learning problem (single task) within a larger learning system encompassing multiple tasks, each with its unique goal and dataset. Fig. 1 illustrates the differences between traditional ML and LML learning (Wang et al 2022a), whereas Fig. 2 illustrates the LML architecture in greater detail (Chen and Liu 2018). LML systems gradually accumulate knowledge from previous tasks and adapt to new tasks whenever they are introduced. This flexibility and the ability to learn sequentially, incrementally, and continually make LML models highly valuable in various research areas and practical applications (Mai et al 2022). When tasks arrive sequentially, this progression may result in class expansion, domain shifts in input, or an increase in task quantity. Considering these aspects are important in KT, as some techniques might not be applicable to all incremental scenarios. Hence, based on these factors, the learning process in LML adheres to three scenarios, encapsulating the sequential, incremental, and continuous aspects of LML (Van de Ven et al 2022):

- *Incremental domain learning (IDL)*: This refers to scenarios where the learner’s structure remains unchanged, but the input data distribution is altered, typically as a result of domain shifts. In IDL, the task identities (Task IDs or Task Boundaries) are not known during testing, and the output space remains the same, as every task employs the same classes.
- *Incremental class learning (ICL)*: ICL is a scenario where a learner progressively learns to recognize more classes by facing tasks that introduce new classes sequentially. It does not require task IDs and is trained to classify all encountered classes. The output’s dimension expands as new classes are introduced, highlighting its ability to generalize across various classes and integrate new ones into its pre-existing knowledge base continuously.
- *Incremental task learning (ITL)*: In ITL, a learner is trained to perform different tasks that have distinct output spaces. The task IDs are known during both training and testing, which allows for having task-specific components like separate output heads or distinct models for each task. *Output heads* refer to the final layer or set of layers in a neural network model that is dedicated to producing task-specific predictions. Each output head is tailored to a particular task, ensuring that learning new tasks does not compromise the model’s performance on previously learned tasks (Hsu et al 2018). The primary goal of ITL is to use shared representations across tasks to optimize computational efficiency and facilitate knowledge transfer between tasks.

In this study, the authors use the term “task” throughout the paper. In the IDL setting, “task” refers to different domains introduced sequentially. When considering ICL, a “task” corresponds to a set of classes introduced together and learned collectively. For ITL, a “task” corresponds to a unique learning problem solved by the learners (Parisi et al 2019; De Lange et al 2022). As such, this study’s terminology aligns with that of the state-of-the-art. However, the reader should note that some studies, including Van de Ven et al (2022), use the term “context” to refer to new domains in IDL and new sets of classes in ICL, while reserving the term “task” for scenarios in ITL where the boundaries of context are known during both evaluation and training.

**Fig. 3:**
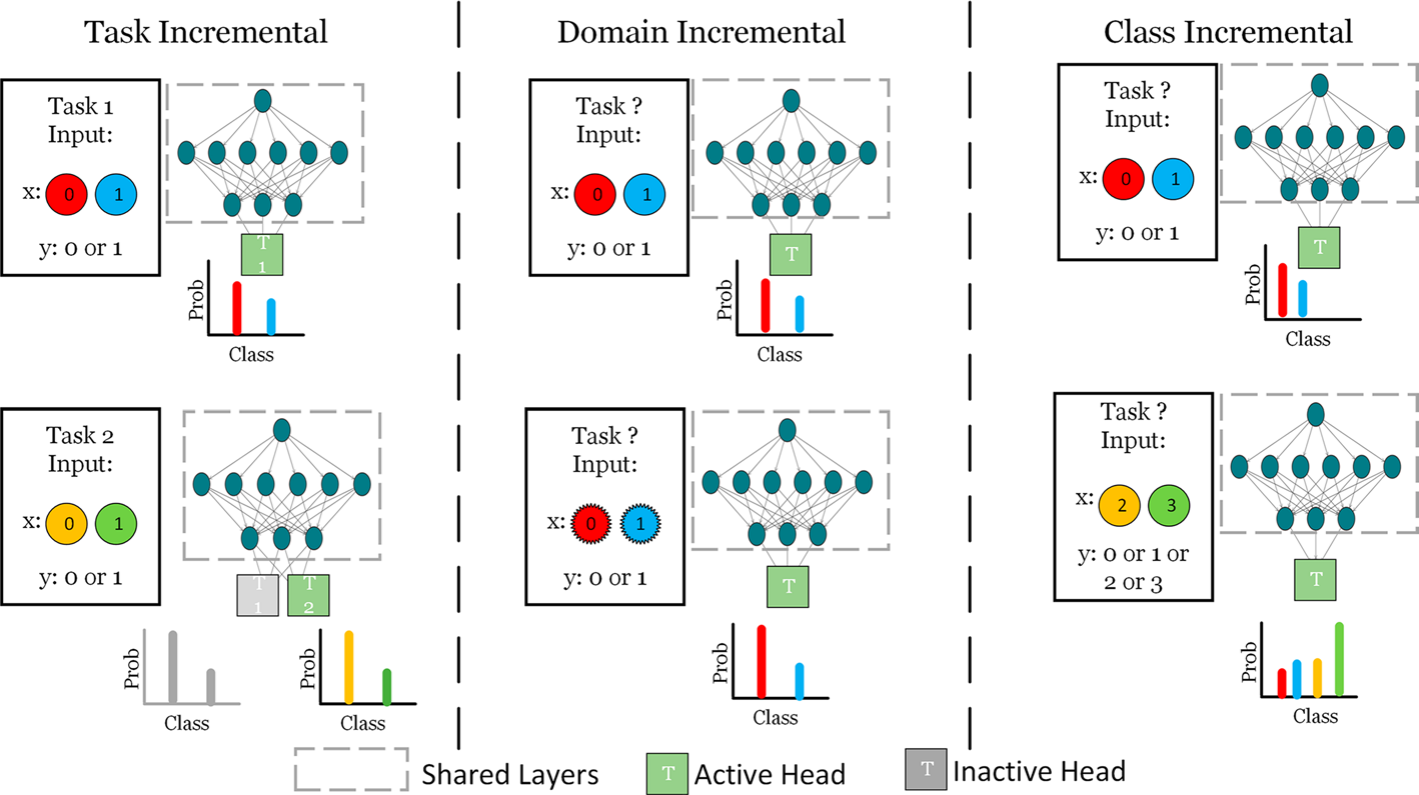
Differences between ITL & IDL & ICL in terms of architecture

Fig. 3 illustrates the distinctions between IDL, ICL, and ITL. In ITL, a new output head is activated with each new task, utilizing shared learner to facilitate KT. Conversely, IDL concentrates on maintaining model performance despite shifts in input representation, depicted in Fig. 3 as noise around the circles. Lastly, in ICL, the model’s output space expands as it identifies new classes. The key distinction between ICL and ITL lies in the accessibility of the Task IDs during both training and testing phases. In ITL, the availability of Task IDs enables the model to leverage task-specific components, thereby enhancing its performance through the effective separation of knowledge relevant to each task. On the other hand, ICL manages an expanding output space to incorporate new classes as tasks are introduced sequentially, with each task potentially contributing a varying number of classes depending on its respective dataset. This expansion necessitates the adaptation of the model’s final layer to include additional classes (e.g., expanding the output space from $y \in \{0, 1\}$ to $y \in \{0, 1, 2, 3\}$ with successive tasks). Conversely, ITL typically responds by integrating new output heads for each new task, catering to a wide array of tasks that can range from classification to regression (e.g., the first head for the initial task is binary classification $y \in \{0,1\}$, while the second head addresses a regression problem $y \in {\mathbb {R}}$). This approach enables ITL models to address a broad spectrum of problems within a cohesive framework, whereas ICL concentrates on incorporating new classes introduced by subsequent tasks. As illustrated in Fig. 3, the T2 head is activated upon the introduction of task two, whereas a single T head is utilized in ICL, expanding to accommodate newly introduced classes.

It’s important to note that LML encompasses various principal aspects, outlined in bullet points below. However, this paper specifically targets KT as it has a crucial role in LML, and given that topics like Catastrophic Forgetting have already been extensively reviewed, as noted by Parisi et al (2019), with other aspects reserved for future research. Following (Kudithipudi et al 2022), the principal areas are categorized as follows:

- *Knowledge Transfer and Adaptation* (Sun et al 2019): Refers to a model’s capability to adjust to new tasks by utilizing the knowledge acquired from earlier tasks (known as forward knowledge transfer), and at the same time, enhancing its understanding of previously learned tasks (termed backward knowledge transfer).
- *Catastrophic Forgetting* (Li et al 2019): Refers to a model’s loss of previously acquired knowledge during the process of learning new tasks. This situation can be compared to replacing old memories with new information.
- *Exploiting Task Similarity* (El Khatib et al 2021): Refers to a model’s ability to share characteristics of old and new tasks to enhance its overall performance.
- *Task-Agnostic Learning* (Van de Ven et al 2022): Refers to a model’s ability to independently identify and adapt to new tasks without needing a signal about the start/end of the task.
- *Noise Tolerance* (Andrychowicz et al 2020): Refers to a model’s ability to work with noisy or inconsistent data, which can happen due to changes in the environment or sensor inaccuracies.
- *Resource Efficiency and Sustainability* (Schwarz et al 2018): Refers to a model’s ability to efficiently manage its memory so only necessary information/data is retained.

In LML, KT is about helping learners keep learning and adapting to new tasks using their acquired knowledge so far. This study refers to chapter 3 of Chen and Liu (2018)’s study, which reveals that the LML community still has not agreed on a single definition of *knowledge*, and does not propose a single definition. Given that new research keeps finding different ways to represent and transfer knowledge, presenting a unified definition of knowledge is challenging. So, in this study, the authors will review existing studies and show how they benefit from KT instead of forming a single definition of knowledge and its transfer technique.

### KT techniques

In this section, the main KT techniques are introduced. The fundamental concepts behind each technique are discussed, with a more detailed analysis reserved for Sect. 5.

#### Regularization

Regularization-based approaches attempt to transfer acquired knowledge by penalizing some neural network weights from being updated (Kirkpatrick et al 2017). These weights have been optimized based on previously seen tasks and represent the knowledge about them. As such, the knowledge will be transferred from one task to the others. Regularization techniques can be either data-focused or prior-focused (Kalb et al 2021), where the former approaches leverage the concept of knowledge distillation (Gou et al 2021). This involves transferring knowledge from a larger teacher model to a small student model; in other words, the loss function is updated based on comparing teacher model outputs, instead of ground-truth labels, with student model outputs (more details are discussed in Sect. 5.1). The latter, prior-focused, concentrates on the weights that are important for previous tasks in order to enhance knowledge transfer. To achieve this, the Regularization technique includes a penalty term in the input-to-output mapping function that discourages significant changes in the model parameters, which are important for the previously learned tasks (Kirkpatrick et al 2017). Regularization-based techniques in data privacy stand out because they avoid storing the original dataset (Gunasekara et al 2022), offering an added privacy advantage. They are also computationally efficient, as they do not necessitate enlarging neural network architectures or preserving training data for future model retraining.

#### Replay

Replay-based or Rehearsal-based techniques work by storing samples from previous tasks in a buffer (e.g., the knowledge-based component of LML) during the training of previous tasks, and replaying them during training on new tasks (Qu et al 2021). In other words, the model will be trained using a combination of stored samples and new task-related data. This enables the model to implicitly incorporate knowledge from previous tasks. The effectiveness of this approach depends on several challenges, including the need for a sufficiently large memory capacity, the ability to collect useful samples, and the ability to store real samples or suitable pseudo-instances (Yang et al 2022). The Replay can be divided into two subcategories, Rehearsal and Pseudo Rehearsal, where the former happens if some part of the original input dataset is stored with respect to the available memory and replayed with a combination of the new task datasets. In pseudo-rehearsal, which is also known as generative Replay, various techniques are used to produce artificial examples, also known as pseudo samples, that represent the original samples, which facilitate knowledge transfer, where the original samples cannot be stored due to privacy matters. For instance, Generative Models like Generative Adversarial Networks (Zhai et al 2019) and Variational Autoencoders (Egorov et al 2021) generate new, realistic data points reflecting original datasets from past learning tasks.

#### Parameter isolation

Parameter Isolation works by freezing selected model parameters or keeping a copy of the entire model for each old task while allowing other parameters or new models to be adapted to new tasks (Aljundi et al 2017; Zhang et al 2023). Parameter Isolation can involve either a fixed or dynamic architecture (Qu et al 2021). In the fixed architecture approach, a single, unchanging architecture is used, where certain parameters, known as task-specific parameters, retain knowledge from previous tasks and remain constant. Meanwhile, other parameters, referred to as shared parameters, begin adapting the model for the new task. The selection of these parameters, whether they are task-specific or shared, can be viewed as an optimization problem. This process aims to identify the most effective configuration within the neural network’s architecture for each specific task. For example, a pathway from the input layer to the output layer is randomly chosen. Based on the requirements of each task, the optimal path is determined and then fixed, or ‘frozen’, for that specific task, as detailed in (Rajasegaran et al 2019). This is akin to utilizing previously acquired knowledge to facilitate learning of the new task. On the other hand, the architecture of neural networks is expanded in the scenarios of dynamic. This implies that additional parameters are incorporated into the existing architecture when a new task is introduced. Consequently, these new parameters are adapted to the new task. In dynamic scenarios, some studies take a different approach (Aljundi et al 2017). Rather than enlarging the parameter set of a single neural network model, this method introduces a distinct model for each task. Additionally, there is a gating mechanism that determines which of the prior models, corresponding to earlier tasks, can be utilized in learning new tasks. This decision is based on the similarities between the previous tasks and the new task. This approach is efficient because it avoids starting from scratch when learning a new task, instead leveraging the knowledge from previous tasks to help in the learning of the new task.

## Literature review methodology

In this section, a detailed explanation is provided regarding the process of formulating a comprehensive search query that covers the research questions, available techniques, evaluation metrics, dataset, and potential future challenges. Additionally, the criteria for including and excluding papers, as well as the quality assessment approach, are discussed.

### Search strategy

The search methodology commenced with an online database search, focusing on *Lifelong Machine Learning* as the primary keyword. This approach led to the identification of a survey paper by De Lange et al (2022). Analysis of this paper, coupled with the authors’ insights, revealed a literature gap concerning KT. In this regard, the keywords “Lifelong Machine Learning” and “Knowledge Transfer” were employed in database searches, utilizing Boolean Operators (AND, OR, NOT) to refine the search. However, this method retrieved irrelevant papers, underscoring the necessity for a more targeted search approach.

The works of Parisi et al (2019) and De Lange et al (2022) were instrumental in developing search queries that yielded more focused and pertinent results in this study’s online database search strategy. Consequently, more specialized terms such as Transfer Learning, Fine-tuning, Regularization, Multi-Task Learning, Curriculum Learning, Experience, and Experience Replay were incorporated into the search query to better align with this study’s focus area. As shown in Table 1, a good portion of the synonyms related to “Knowledge Transfer” and “Methods” were derived from these two key studies (7 out of 11), establishing a standard taxonomy and refining the scope of the research.

Using this refined query (Table 1), the authors conducted an **online automated database search strategy** to retrieve papers for further investigation regarding their relevance to this study’s subject. While Parisi et al (2019) provided insights into the biological and machine learning aspects of LML, and De Lange et al (2022) addressed issues of forgetting, the current study delves into the specifics of state-of-the-art KT techniques. The outcomes from each database, based on the refined search query, are discussed in subsequent sections.

### Source databases

In this study, the focus is only on academic sources, and grey literature was excluded from the search process. The following databases were explored using specific search queries in May 2024:

- Scopus
- Web of Science
- IEEE Xplore
- ACM Digital Library

The databases Scopus and Web of Science were selected for their comprehensiveness as they are among the most widely used and trusted sources for academic research. In addition, IEEE Xplore and ACM Digital Library were chosen due to their inclusion of ML articles.

### Abstract search query

This study centers around three core concepts: Lifelong Learning, Knowledge Transfer, and Machine Learning. Table 1 presents the key concepts and their corresponding synonym terms that will be used to formulate the query.

**Table 1:** Terms and corresponding synonyms for search query

Concept	Synonyms	Corresponding Query
Lifelong Learning	$\bullet$ Continual learning	*“Lifelong learning” OR “continual learning” OR “lifelong machine learning”*
	$\bullet$ Lifelong machine learning	
Knowledge Transfer	$\bullet$ Experience	Experience OR ( knowledge PRE/3 transfer* ) OR ( learning PRE/3 transfer* ) OR ( “transfer learning”) OR information
	$\bullet$ Knowledge transfer	
	$\bullet$ Learning transfer	
	$\bullet$ Transfer learning	
	$\bullet$ Information	
Methods	$\bullet$ Fine-tuning	“Fine-tuning”OR “regularization” OR “multi-task learning” OR “curriculum learning” OR “experience replay” OR technique*
	$\bullet$ Regularization	
	$\bullet$ Multi-task learning	
	$\bullet$ Curriculum learning	
	$\bullet$ Experience replay	
	$\bullet$ Technique	
Machine Learning	$\bullet$ Deep learning	*“Machine learning” OR “deep learning” OR “artificial intelligence” OR “neural network”*
	$\bullet$ Artificial intelligence	
	$\bullet$ Neural network	

To align with the research objectives, the focus of this study is confined to the domain of LML. However, due to the overlapping nature of this term with similar terms in education and social studies (Laal et al 2014), the inclusion of Machine Learning and its synonyms is necessary to delimit the scope of this study. This inclusion is emphasized due to the occurrence of terms such as Curriculum Learning, Technique, Experience, and Information within the educational context. The authors’ intent in incorporating Curriculum Learning extends beyond the educational area, aligning instead with another learning paradigm that parallels LML. According to Wang et al (2022a), Curriculum Learning involves a sequential task learning approach, beginning with simpler tasks and progressing to more challenging ones. The concepts of Experience, Information, and Technique are identified as synonymous with knowledge and KT methods, as elucidated by Parisi et al (2019). Thus, the inclusion of terms listed in the *Synonyms* column of the Machine Learning row in Table 1 aids in narrowing the search towards relevant papers in LML area.

Table 1 presents the key concepts, along with their synonyms and the associated search queries. The utilization of the wildcard character "*" allows for the inclusion of various word forms, such as *transferred* and *transferring*. Additionally, the *near/pre* operator has been employed in conjunction with maximum three-word proximity to enhance the specificity of the search results related to KT. Quotation marks have been used in the queries to ensure a focused search on the central concepts. Given the mentioned points, the search queries in different databases are presented in the Appendix, Table 5.

### Exclusion criteria

The following criteria were used to exclude papers:

1. **Relevance**: Papers without a primary focus on techniques for KT in LML were eliminated. This eliminated any paper related to KT in the field of education (e.g., non-AI-related publications). In fields outside AI, lifelong learning emphasizes the continuous development of human abilities in a supportive environment, fostering the acquisition of knowledge, values, and skills for application across various life scenarios (Jarvis 2012). Accordingly, while studies that define their problem within the context of enhancing human learning capacities are not be considered, publications dealing with Lifelong Learning in ML and AI areas are included. Such approach resulted in including the studies analyzing educational outcomes, such as student performance using LML.
2. **Language**: Papers written in languages other than English were not considered.
3. **Peer Reviewed**: Any papers that were not peer-reviewed, such as preprinted papers, posters, short papers, abstracts, reports, theses, and book chapters, were not taken into consideration.
4. **Survey Papers**: Any review papers that do not add insight toward KT in LML were excluded.

### Inclusion criteria

For the papers to be included in the study, the following criteria must be met:

- **Relevance**: Papers focused on KT in LML were moved for the full-text screening process.
- **Methodological Rigor**: Only papers describing a well-designed experimental setup, analysis methods, and clear statements of results were moved for the full-text screening process.

It is important to note that the screening process was conducted with the help of the Covidence^1^ tool. Initially, Research Information Systems (RIS) files were extracted from databases using specific search queries. Subsequently, these RIS files from four databases were imported into Covidence, where duplicates were automatically eliminated. The remaining papers underwent abstract screening, with the authors employing a voting approach to determine whether each paper met the inclusion and exclusion criteria for the full screening phase. Finally, details of papers selected for full screening were extracted from Covidence, and the authors applied their methodology for information extraction during the full review process.

### Search results

**Fig. 4:**
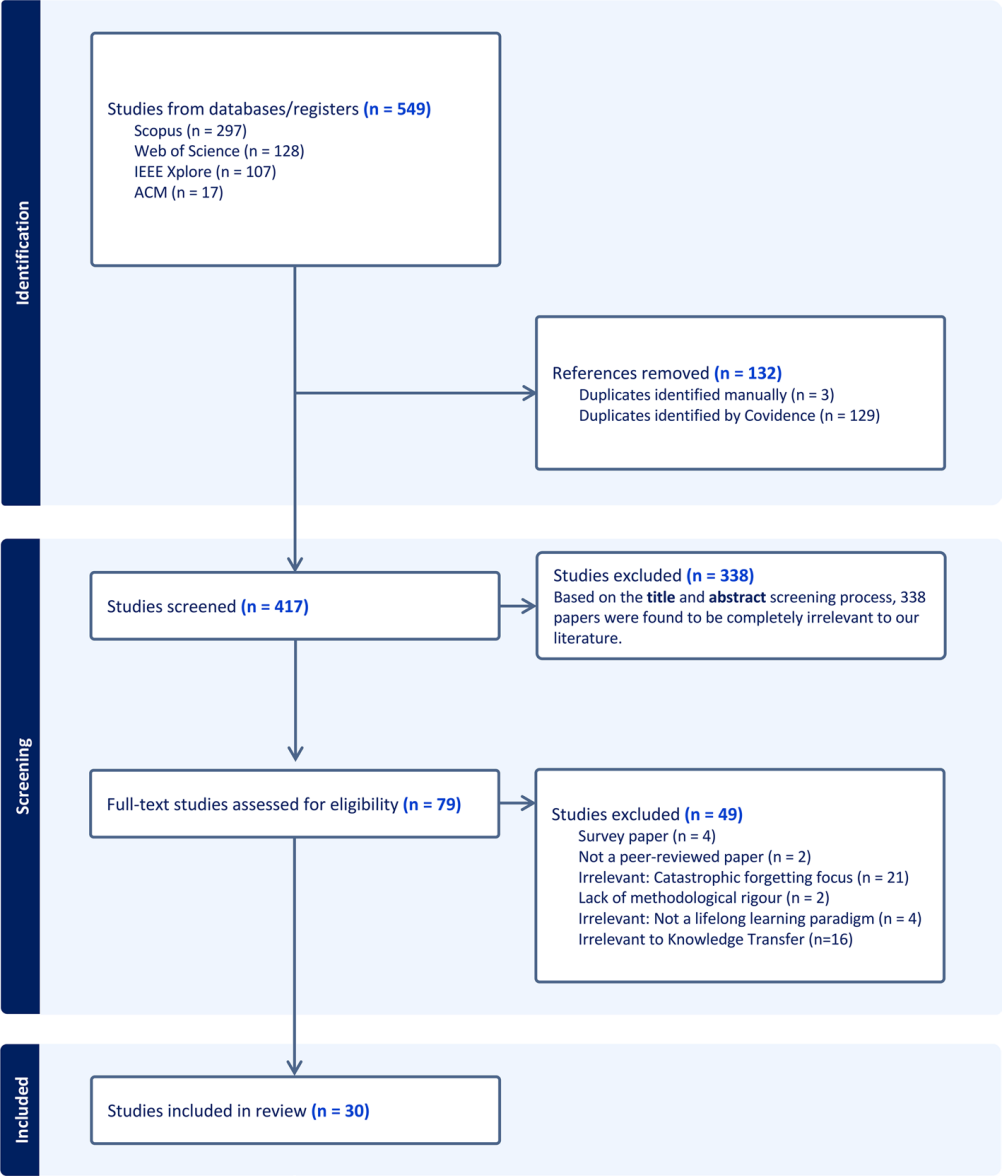
PRISMA diagram obtained from covidence

Fig. 4 shows the publications retrieved through the search query that meet inclusion criteria. As shown in Fig. 4, databases such as Scopus and Web of Science yield a higher number of papers than IEEE Xplore and ACM Digital Library. In view of the paucity of search tools that can simultaneously process titles, abstracts, and keywords in the ACM Digital Library, the authors had to restrict the search to abstracts exclusively, leading to a reduction in the number of papers available. In total, 549 papers were obtained from the databases, but after eliminating duplicates, the number of papers for review was reduced to 417 (Fig. 4). As depicted in Fig. 4, the initial set of 417 papers was narrowed down. After screening the abstracts and titles of the initial set of papers, the authors identified 338 studies that were deemed irrelevant to this study’s focus. From the remaining 79 papers, 49 were excluded during the full-text screening review stage due to the exclusion and inclusion criteria represented in Fig. 4. Consequently, 30 studies were ultimately included in this review to extract the information.

## Extracted data from the selected papers

In line with the RQ1 or RQ2, the following data were extracted:

### Extracted data for RQ1

The data presented in each column of Table 2 were selected to address the first research question. Each column presents:

- **Article**: Authors’ names and publication dates to facilitate the identification of the selected papers.
- **Learning Type**: Category of ML, namely Supervised Learning, Unsupervised Learning, and Reinforcement Learning. The inclusion of this column aims to facilitate the identification of the paradigm that has made greater use of LML’s capabilities.
- **Task/Domain/Class (T/D/C) Incremental learning**: Each study regarding the incremental learning approach has a central idea represented by this column. Section 2 reviewed the attributes of Incremental Task Learning (ITL), Incremental Domain Learning (IDL), and Incremental Class Learning (ICL).
- **KT Technique**: In this column, the authors represent each study’s chosen knowledge transfer technique. The options are Replay, Regularization, and Parameter Isolation.
- **Modeling Paradigm**: Underlying modelling paradigm of the studies, which can be either *neural network (NN)* or specific branch of *non-neural network (non-NN)*.
- **Architecture**: Main architectures employed in each publication: static and dynamic. By “static,” the authors mean studies in which the introduction of new tasks does not result in the expansion of parameters or the adding of new models to a pool of models. Conversely, “dynamic” refers to scenarios where such expansion and storage are possible.
- **Statistical Test (S Test)**: A crucial aspect of current research involves validating claims about model output quality through statistical testing. This column helps us figure out the mentioned investigation aspect in the selected papers.
- **Code**: The availability of code, which can be accessed via GitHub^2^. The inclusion of this information is of great importance as it promotes reproducibility and facilitates future research in the field.

**Table 2:** Information collected to address RQ1

Article	Learning Type	T/D/C	KT Technique	Modeling Paradigm	Architecture	S Test	Code
Ruvolo and Eaton (2013)	Supervised Learning	ITL	Parameter Isolation	Non-NN Logistic and Simple Regression	Dynamic	No	No
Khan and Khalid (2017)	Unsupervised Learning	IDL	Parameter Isolation	Non-NN Paradigmatic and Syntagmatic Rule	Dynamic	No	No
Tessler et al (2017)	Reinforcement Learning	ITL & IDL	Replay & Parameter Isolation	Neural Network	Dynamic	No	No
Clingerman and Eaton (2017)	Supervised Learning	ITL	Parameter Isolation	Non-NN Gaussian Processes	Dynamic	No	No
Sun et al (2018)	Supervised Learning	ITL	Parameter Isolation	Non-NN Logistic and Simple Regression	Dynamic	No	No
Riemer et al (2019)	Supervised/ Reinforcement Learning	IDL & ITL	Replay	Neural Network	Dynamic	No	GitHub$^{3}$
Doyle et al (2019)	Reinforcement Learning	ITL	Parameters Isolation	Neural Network	Static	No	No
Benavides-Prado et al (2020)	Supervised Learning	ITL	Parameter Isolation	Non-NN Support Vector Machine	Dynamic	No	No
Gupta et al (2020)	Unsupervised Learning	IDL	Replay & Regularization	Neural Network	Static	No	GitHub$^{4}$
Pandit and Kudithipudi (2020)	Reinforcement Learning	ITL	Parameters Isolation	Neural Network	Dynamic	No	No
Ke et al (2021)	Supervised Learning	ITL	Parameters Isolation	Neural Network	Dynamic	Paired t-test	GitHub$^{5}$
Li et al (2021)	Reinforcement Learning	ITL	Replay	Neural Network	Static	No	No
Korycki and Krawczyk (2021)	Supervised Learning	ICL	Replay	Neural Network	Static	No	GitHub$^{6}$
Mahmoud and Hajj (2022)	Supervised Learning	ITL	Regularization	Neural Network	Static	No	No
Yang et al (2022)	Supervised Learning	IDL & ITL	Replay	Neural Network	Static	No	No
Cai et al (2022)	Supervised Learning	ITL & ICL	Regularization & Parameter Isolation	Neural Network	Dynamic	No	No
Han and Liu (2022)	Supervised Learning	ITL	Replay & Parameters Isolation	Neural Network	Static	No	No
Gautam et al (2022)	Supervised Learning	ICL	Replay & Regularization	Neural Network	Static	No	GitHub$^{7}$
Gao et al (2022)	Supervised Learning	ITL	Parameters Isolation	Neural Network	Dynamic	No	GitHub$^{8}$
Mei et al (2022)	Supervised Learning	ITL	Parameters Isolation	Neural Network	Static	No	GitHub$^{9}$
Sun et al (2022)	Supervised Learning	ICL & ITL	Parameters Isolation	Neural Network	Dynamic	No	No
Zhao et al (2022)	Unsupervised Learning	IDL	Replay	Neural Network	Dynamic	Friedman Test & Wilcoxon Signed-Rank Test	GitHub$^{10}$
Zaman et al (2023)	Supervised/ Unsupervised Learning	ITL	Parameters Isolation	Neural Network	Dynamic	No	No
Li et al (2023)	Supervised Learning	IDL & ITL	Replay & Regularization	Neural Network	Dynamic	No	No
Boschini et al (2023)	Supervised Learning	ICL & ITL	Replay & Regularization	Neural Network	Dynamic	No	GitHub$^{11}$
Tian et al (2023)	Supervised Learning	ICL & ITL	Replay & Regularization	Neural Network	Dynamic	No	GitHub$^{12}$
Kozal and Wozniak (2023)	Supervised Learning	ITL	Parameters Isolation	Neural Network	Dynamic	No	GitHub$^{13}$
Chen et al (2023)	Supervised Learning	ITL	Regularization & Parameter Isolation	Neural Network	Dynamic	No	No
Li et al (2024)	Supervised Learning	ICL	Replay	Neural Network	Static	No	No
Yu et al (2024)	Supervised Learning	ICL	Replay	Neural Network	Static	No	No

$^{3}$https://github.com/mattriemer/mer

$^{4}$https://github.com/pgcool/Lifelong-Neural-Topic-Modeling

$^{5}$https://github.com/ZixuanKe/LifelongSentClass

$^{6}$https://github.com/lkorycki/rsb

$^{7}$https://github.com/Chandan-IITI/Tf-GCZSL

$^{8}$https://github.com/gcooq/CLEAS

$^{9}$https://github.com/zylMozart/TaskDrop

$^{10}$https://github.com/KingSpencer/DBULL

$^{11}$https://github.com/aimagelab/mammoth

$^{12}$https://github.com/TianSongS/PMKD-IL

$^{13}$https://github.com/w4k2/increasing-depth-life-long

### Extracted data for RQ2

Information extracted to answer this question is presented in Table 3. Each column of the table contains the following information:

- **Metric for evaluating**: List of the metrics considered in the study.
- **Utilized dataset**: Name of the dataset used in the study.

**Table 3:** Information collected to address RQ2

Article	Metric for Evaluating	Utilized Dataset
Ruvolo and Eaton (2013)	AUC & ROC & RMSE	Synthetic Regression Task & London School & Land Mine Detection & Facial Expression Recognition
Khan and Khalid (2017)	Topic Coherence	Amazon Review Dataset
Tessler et al (2017)	Reward	Minecraft
Clingerman and Eaton (2017)	Time & RMSE	Synthetic Regression Task & London School & Robot Arm Kinematics & Parkinson’s Vocal Tests
Sun et al (2018)	AUC & RMSE	Disjoint & London School & Parkinson’s Vocal Tests & Smart Meter & Landmine & CUB-200
Riemer et al (2019)	RA & LA & BWT	MNIST Permutation & MNIST Rotation & Omniglot & Catcher & Flappy Bird
Doyle et al (2019)	Reward	Grid World
Benavides-Prado et al (2020)	CGLL & Average Accuracy	20Newsgroup & CIFAR-100 & ImageNet
Gupta et al (2020)	Recall @ Precision & Topic Coherence & PPL	Agnews & Topic Memory Networks (TMN) & R21578 & 20Newsgroup
Pandit and Kudithipudi (2020)	Reward	Simulated Environment
Ke et al (2021)	Average Accuracy & Individual Accuracy	Amazon Review Dataset
Li et al (2021)	Reward	StarCraft & Gird World
Korycki and Krawczyk (2021)	Normalized Accuracy & Average Accuracy	FMNIST & MNIST & CIFAR-10 & ImageNet & SVHN
Mahmoud and Hajj (2022)	Average Accuracy	ALKAN & DAS & OPPORTUNITY & HAR
Yang et al (2022)	AER & FGT & ADE & FDE	ETH & SDD & UCY
Cai et al (2022)	FGT & Average Accuracy	Amazon Review Dataset
Han and Liu (2022)	Average Accuracy & Forgetting Measure & LA	CIFAR-10 & CIFAR-100 & SVHN
Gautam et al (2022)	mSA & mUA & mH	CUB-200 & aPY & Animals with Attributes & SUN
Gao et al (2022)	Average Accuracy & Model Complexity & Number of Parameters	MNIST Permutation & MNIST Rotation & CIFAR-100
Mei et al (2022)	Transfer Accuracy & Mutual Transfer	Amazon Review Dataset
Sun et al (2022)	AUC & RMSE & Time	Disjoint & London School & Parkinson’s Vocal Tests & Smart Meter & Landmine & CUB-200
Zhao et al (2022)	NMI & ARS & HS & CS & VM	MNIST & STL-10 & Reuters10K
Zaman et al (2023)	Recall & Precision & Average Accuracy & F1-Score & Inlier Ratio & RFR & FMR	Oxford & 3D Match & KITTI & ETH
Li et al (2023)	Average Accuracy & BWT	MNIST Permutation & MNIST Rotation & SVHN & MNIST & USPS & CIFAR-10 & CIFAR-100 & Tiny ImageNet
Boschini et al (2023)	Average Accuracy & Forgetting Measure & AUC	CIFAR-100 & Tiny ImageNet & NTU-60
Tian et al (2023)	Average Accuracy & Forgetting Measure	CIFAR-10 & CIFAR-100 & CUB-200 & ImageNet
Kozal and Wozniak (2023)	Average Accuracy & Forgetting Measure	CIFAR-100 & Tiny ImageNet & CORe50
Chen et al (2023)	Average Accuracy & F1 Score & Perplexity	Wikipedia
Li et al (2024)	Average Accuracy & Forgetting Measure & BWT & FWT	MNIST & FMNIST & CIFAR-10 & CIFAR-100 & Tiny ImageNet
Yu et al (2024)	Average Accuracy & Forgetting Measure	CIFAR-10 & CIFAR-100 & Tiny ImageNet & mini ImageNet

*Bold texts in Metric for Evaluating column demonstrates the newly introduced metrics

## Results

This section starts with detailing the various KT techniques used in the retrieved papers. Subsequently, metrics and datasets used in the selected papers are listed.

### Techniques for KT

**Fig. 5:**
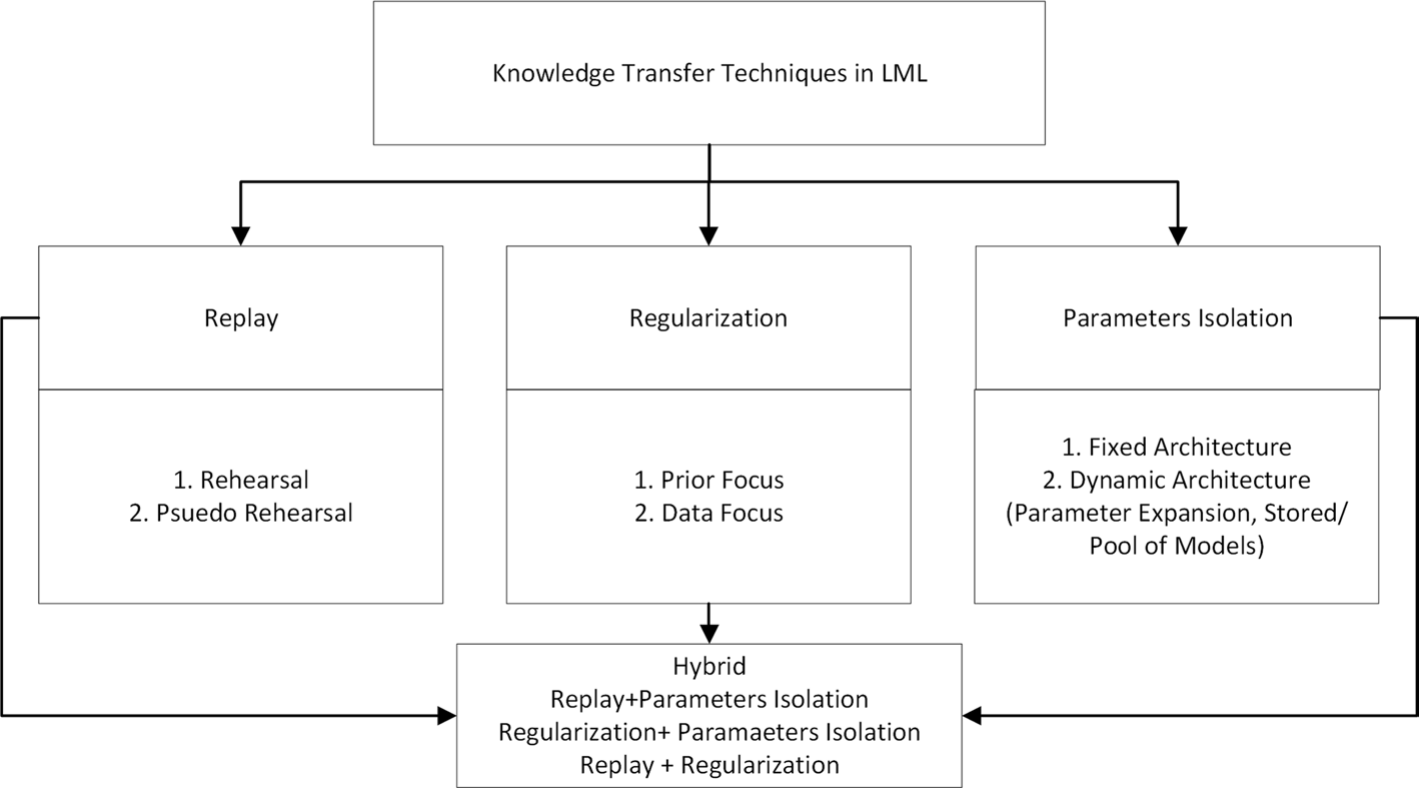
Result section’s structure for analyzing KT in LML

As shown in Table 2 KT techniques are categorized into Replay, Parameter Isolation, Regularization, or Hybrid, following the established categories from De Lange et al (2022). Fig. 5 illustrates how the discussion of KT techniques will be structured in the upcoming subsections.

#### Regularization

As discussed in Sect. 2.2, the Regularization technique can be either data- or prior-focused.

***Prior-Focused:*** Prior-focused Regularization aims to keep the knowledge associated with previous tasks from being diminished by incorporating a penalty term into the neural network’s mapping function. This term is designed to discourage significant changes in the model parameters that are important for the tasks learned previously, as outlined in Kirkpatrick et al (2017). To determine the importance of these parameters for each task, Elastic Weight Consolidation (EWC), a model based on prior-focused Regularization, utilizes the Fisher Information Matrix, as discussed in Huszár (2018). A straightforward method to prevent important parameters from straying too far from the values optimized for previously learned tasks is through L2 Regularization. L2 Regularization operates by adding a penalty term to the loss function, which is proportional to the sum of the squares of the model’s weights.

***Data-Focused:*** Regarding data-focused, this involves transferring knowledge from a larger teacher model to a small student model by using the teacher’s outputs as soft labels^3^ in training the student model. The Learning without Forgetting (LwF) approach, which is a data-focused Regularization study, involves using a previously trained model as a teacher to help a student model learn new training data without forgetting the old information (Li and Hoiem 2018).

The study by Mahmoud and Hajj (2022) diverges from the commonly explored image-based studies in LML, focusing instead on time-series data. This research utilizes a multi-objective loss function to improve knowledge transfer, which is essential in addressing shifts in both class label and input distribution. In their setup, they applied different penalties to three losses (Knowledge distillation, Autoencoder Reconstruction, and classification Cross-entropy) in order to improve the likelihood of transferring important information. Knowledge Distillation (KD) utilizes a model trained on old tasks as a pre-trained model (i.e., teacher model) and transfers knowledge to a student model for a new task through soft labels. KD loss fosters backward knowledge transfer by penalizing parameters that lead to decreased performance in the teacher model (i.e., the source domain). In contrast, cross-entropy loss aids the model in accurately predicting the ground-truth label as compared to the soft label used in KD. This is achieved by penalizing misclassification predictions and adding a penalty term to the cross-entropy loss. Autoencoder Reconstruction (AR) loss addresses shifts in the input feature distribution that occur over time and across tasks in time series data. Incorporating AR loss with a penalty term into the overall loss function helps the proposed model to maintain its generalization capability and share mutual knowledge in response to shifts in the input feature distribution. Their proposed solution, the Lifelong Learning Multi-task Autoencoder (LOMA), leverages a hierarchical convolutional recurrent architecture, making it adaptable to time-series tasks. In their approach, Mahmoud and Hajj (2022) prioritize beginning with the task that demonstrates the best performance in sequential learning, especially when there are multiple tasks (more than two) to be learned. Four time series benchmarks based on human activity recognition were used to evaluate the model’s performance.

#### Replay

As stated in Sect. 2.2, rehearsal (Replay) and pseudo rehearsal (generative Replay) are two main sub-categories of Replay technique.

***Rehearsal/Replay***: *“Incremental Classifier and Representation Learning”* (iCaRL) as a rehearsal approach designed to incrementally learn new classes while preserving knowledge of previously learned ones (Rebuffi et al 2017). It achieves this by continuously updating the feature mapping function for all classes and employing a nearest-mean-of-exemplars classifier, rather than a traditional softmax layer, for classification. Exemplar management is a crucial part of iCaRL, where the randomly selected set of representative examples for each class is stored to balance memory usage depending on the computational limitation. iCaRL treats every class equally, assigning the same number of representative samples to both major and minor classes. These exemplars are used for rehearsal, a process where the model is periodically retrained on a mixture of new data and old exemplars to transfer knowledge.

The study by Riemer et al (2019) aims to adjust the gradients of neural network weights across instances using a trade-off to maximize transfer and minimize interference between the task. The method is designed to make interference (the negative impact of new information on previously learned knowledge) less likely based on future gradients while simultaneously making transfer (the advantages of previously learned knowledge to new tasks) more likely. In this context, the role of stored samples becomes crucial. By analyzing the gradients of stored samples alongside current task data within a batch, the objective is to update the reservoir of stored samples. This update process involves retaining samples that facilitate knowledge transfer (indicated by positive gradients) and discarding those that heighten the risk of forgetting (indicated by negative gradients). They propose Meta-Experience Replay (MER) to modify the gradients and increase the probability of achieving the mentioned trade-off. One of the notable strengths of their work is that they consider both supervised and reinforcement learning paradigms and show that their proposed MER method can perform comparably well in both scenarios.

Korycki and Krawczyk (2021) present an innovative approach for KT in LML under concept drift conditions. The paper introduces a system that combines Experience Replay with a centroid-driven memory for class-based prototypes and a reactive subspace buffer (RSB). Class-based prototypes are representative original samples of each class, serving as a compact summary of the class features. The centroid-driven memory manages these prototypes by maintaining the centroids of each class in the memory. This system dynamically tracks concept drifts in classes, allowing for adaptive clustering and memory updates for having better representative samples in the buffer that are replayed in learning new tasks to transfer the knowledge. Centroid-driven memory distinguishes between retaining useful knowledge and discarding outdated information, ensuring efficient and relevant information is used for KT, the process which is known as RSB. This process is done by comparing the centroid of each class and ongoing task’s data. The methodology effectively balances between remembering valid information and adapting to new or evolving concepts.

Li et al (2024) propose “AdaER: An Adaptive Experience Replay Approach for Continual Lifelong Learning,”. In this paper, they propose a novel approach to enhance knowledge transfer by using two key strategies: Contextually-Cued Memory Recall (C-CMR) and Entropy-Balanced Reservoir Sampling (E-BRS). C-CMR aims to select the most representative samples for Experience Replay by maintaining two types of buffers: an example-interfered buffer ($R_e$) and a task-associated buffer ($R_t$). The example-interfered buffer $R_e$ stores samples that exhibit high levels of interference with the current task, identified by a virtual classifier’s (trained only on the current task without Replay) performance drop on these samples compared to the current model. The task-associated buffer $R_t$ includes samples from tasks most impacted by interference but not necessarily showing the highest forgetting degree. By combining $R_e$ and $R_t$, C-CMR effectively selects samples that help maintain model performance and generalization across all tasks. Additionally, E-BRS enhances the memory update process by optimizing the information entropy of the Replay buffer, ensuring a balanced representation of all classes, especially in imbalanced data scenarios. This method replaces the least informative samples in the most overrepresented classes, preserving essential information and reducing bias. The AdaER algorithm thus leverages these strategies to adaptively select and update memory samples, addressing KT and improving lifelong learning performance.

Yu et al (2024) introduces a novel method called "Contrastive Correlation Preserving Replay (CCPR)" for addressing enhancing KT in online continual learning. CCPR enhances the model’s ability to maintain knowledge across tasks by preserving the correlation structures among learned representations. This is achieved using a correlation-preserving memory update mechanism where the model updates its memory buffer not just by storing raw data points, but by ensuring that the relational information between features is maintained. This mechanism leverages mutual information maximization, ensuring that the correlation in features between the current task and the stored memories is preserved. Importantly, the method employs a contrastive loss function that minimizes the distance between representations of similar samples while maximizing the distance between dissimilar ones, fostering a robust feature space that better generalizes across tasks. This approach not only reinforces the relevant connections between past and current knowledge but also dynamically manages memory to balance between retaining old informational structures and accommodating new insights, effectively stabilizing learning over successive tasks.

***Pseudo Rehearsal/Generative Replay***: In the paper *“Self-generated Long-term Experience Replay for Continual Reinforcement Learning”* (Li et al 2021), the methodology centers around reinforcement learning in dynamic environments like StarCraft II, a real-time strategy game, and GridWorld, a navigation task environment. Actions in these contexts are decisions made by the agent (e.g., moves like left, right, up, and down or attacking), while states represent the different scenarios in the environment (e.g., a state could be a specific configuration of units on the battlefield, and an action would be ordering a unit to attack). The rewards are feedback based on the agent’s actions, guiding it toward optimal behaviour. The innovation of the Self-generated Long-term Experience Replay (SLER) mechanism lies in its unique approach to handling learning tasks in a continual learning environment. The SLER mechanism cleverly divides responsibilities between two models: *"the Model Learner and the Replay Generator"*. Model Learner’s Role is the dynamic learner, constantly adapting to new information. It focuses exclusively on learning from new states ($S_{new}$) and actions ($A_{new}$). Whenever a new task is introduced, the Model Learner resets, allowing it to focus entirely on the latest task without interference from previous tasks’ data. In contrast, the Replay Generator is the memory keeper. It does not reset with each new task. Instead, it continuously generates simulated states and actions based on a model that is trained on both new task samples and old simulated experiences from the past ($S_{new \& old}$, $A_{new \& old}$). This process creates pseudo samples, simulated states, to be used for learning along with the new task’s dataset, which helps in transferring knowledge and learning new tasks while not losing performance on previous tasks. This approach involves storing initial states, actions, and rewards, then using the model to recreate intermediate states for Replay, thus helping knowledge transfer in Multi-Task Learning environments.

Zhao et al (2022) suggest *“Deep Bayesian Unsupervised Lifelong Learning”* to deal with the issue of transferring knowledge in unsupervised lifelong learning (ULL). The method, known as Deep Bayesian ULL (DBULL), uses a fully Bayesian inference framework to learn from continuous streams of unlabelled data. It introduces an innovative way of preserving knowledge by using sufficient statistics of the latent representation (encoded samples) of raw data. DBULL operates by using latent representations to capture key features of data in a compressed format that saves memory by accumulating pseudo samples (generated encoded samples) instead of original samples. These representation samples are updated/replaced using sufficient statistics that represent the higher probability of an example being a member of a specific cluster. Sufficient statistics represent the probability of a sample belonging to a specific cluster based on Gaussian distribution. Additionally, DBULL has a unique feature for automatically discovering clusters and eliminating redundancy. The system dynamically forms new clusters as fresh data comes in, enabling the model to adjust to evolving data patterns. It also merges clusters when they show almost similar statistics, keeping a clean and efficient representation of the knowledge it has learned. This method is beneficial for saving memory space and quickly adapting to new data.

“*The Continual Learning-based Trajectory Prediction with Memory-Augmented Networks*” (CLTP-MAN) study, as detailed by Yang et al (2022), employs a combination of rehearsal and pseudo-rehearsal techniques. The study’s framework includes three primary modules: 1) external memory, 2) memory extraction, and 3) trajectory prediction, along with an additional component termed Coreset. Each new task is used to train the memory extraction module, based on an autoencoder, to encode and decode knowledge into the external memory, thereby optimizing memory use. The embedded inputs (pseudo samples) are stored in external memory, guided by a controller, a part of the memory extraction module, trained based on embedding features. This controller discerns which samples to add (write) and when to retrieve (read) stored samples for new tasks. The samples here are related to the pedestrian trajectory patterns. At the end of each task, 1% of the original samples are preserved in the Coreset. The trajectory prediction module, trained on the Coreset and decoded samples chosen by the controller, then makes predictions. With each subsequent task, this process repeats, considering previous samples from the Coreset. The study employs multimodal trajectory data; by multimodal, the authors refer to the possibility of multiple pedestrian trajectory patterns deriving from decoding external memory. It reveals that generated trajectories deviate from ground-truth trajectories after learning the second and afterward tasks, as the knowledge (i.e., samples and pseudo samples) from the first and previous tasks impact the trajectory generation for subsequent tasks.

Among the studies that were examined, Replay methods have consistently demonstrated remarkable performance. The primary reason behind their effectiveness lies in the presence of a continuously refreshed buffer containing samples from previously learned tasks. When the model undergoes retraining, it is more likely to transfer vital information from its past experiences (Korycki and Krawczyk 2021). Nonetheless, Replay methods do face some enduring challenges. As the Replay methods need to store original samples from the dataset, they are not a good candidate for the application of healthcare (Armstrong and Clifton 2022) and computer vision (Hassanpour et al 2022). In addressing privacy concerns, certain studies have leveraged the strategy of storing pseudo samples within a buffer for future Replay (Wang et al 2022b). This innovative approach has helped Replay methods remain relevant in scenarios where privacy constraints are in place.

#### Parameter isolation

Parameter Isolation technique is discussed considering two subcategories: (1) Fixed and (2) Dynamic Architectures.

***Parameter Isolation with Fixed Architecture***: Doyle et al (2019) introduce a method for lifelong learning in reinforcement learning environments, named *"Variational Policy Chaining (VPC)"*. This method facilitates knowledge transfer by creating a unified policy chain (mapping function: states to the optimal actions) from all previously learned policies. The creation of the policy chain occurs as follows: In each training session, the policy chain begins to learn a new task. Upon completion, there are two policies: the prior policy chain, which represents the accumulated knowledge before the new task, and the posterior policy, a modification of the prior policy chain adapted to the new task. Subsequently, a Kullback–Leibler divergence-based loss function starts to minimize the difference between the distributions of the prior policy chain and the posterior policy to adjust the parameters in a way that shares more common knowledge about all previous tasks. This minimization is achieved through gradient descent calculations, effectively reducing the distance between the two conditional distributions - the prior policy chain and the posterior policy. This optimized policy chain serves as a starting point for new tasks, enhancing both forward and backward knowledge transfer (KT). It evolves with each new task, ensuring a more efficient and effective learning process in lifelong reinforcement learning scenarios. This study focused on shared parameter learning rather than freezing parameters for task-specific learning by training a single variational policy chain sequentially.

Mei et al (2022) introduces TaskDrop, which uses random binary masks for each task instead of the traditional method of Parameter Isolation, where always one fixed part of the model was responsible for learning a specific task. These masks are simple binary arrays that selectively activate or deactivate parts of the model (group of parameters) for being trained considering distinct tasks. What sets TaskDrop apart is its flexibility; it allows these task-specific parameters to change during updates and does not make them completely frozen. This method strikes a balance between learning new tasks and maintaining previously acquired knowledge by accepting some degree of reduction in KT to accommodate new learning.

***Parameter Isolation with Dynamic Architecture***: In the paper by Zaman et al (2023), the proposed ***"Continual Multiplex Dynamic Graph Attention Network"*** (CMDGAT) addresses the challenge of accurately matching 3D points in sequential scans for 3D scene reconstruction and augmented reality given the complexities such as dynamic objects, overlapping regions, and changing viewpoints. This approach involves three main components: (1) Keypoint Encoding, where an encoder begins by encoding keypoints, which are significant features like edges and corners within point clouds. A keypoint encoder transforms these points and the raw point clouds into high-dimensional vectors containing descriptive information and passes them to CMGAT. (2) The heart of the system is CMDGAT, which is composed of two interconnected models - the *"Knowledge Model (KM) and the Live Model (LM)."* This network utilizes self-attention for processing within a single point cloud (intra-cloud) and cross-attention for linking multiple clouds (inter-cloud) to recognize parameters that are related to learning keypoints of new tasks. This dual attention mechanism refines the keypoint descriptors. The KM stores parameters for previous tasks by adding a layer with an attention block for each new task. The LM, connected to the last layer of KM in a feed-forward way, adapts to new tasks by accessing KM’s layers and applying self and cross-attention to understand keypoints of new tasks in relation to previously learned tasks. (3) The final step employs an Association Layer, which uses the enhanced descriptors from CMDGAT to calculate a score matrix. This matrix evaluates relationships or similarities among keypoints, aiding in the partial association representation between them.

*“Flexible Clustered Lifelong Learning”* (FCL$^3$) is a developed approach in LML introduced by Sun et al (2022). It is also categorized as Parameter Isolation with dynamic architecture. FCL$^3$ is designed with two main parts: a *“feature learning library and a model knowledge library”*. The feature learning library uses an autoencoder, which is a type of neural network for reconstructing the inputs. The feature library holds the autoencoder learned from all the previous tasks. This is useful because it enables the system to calculate the loss value by reconstructing the input using the available autoencoder and current task as input. Whenever a new task comes up, the system decides whether it is similar to tasks it already knows or is very different by calculating the loss value in the autoencoder. If the task has a lower loss, the system improves its autoencoder (feature library) and uses the stored representative models based on the probability of being useful models for ongoing tasks assigned by alternating direction method of multipliers (Boyd et al 2011). But if the task has a higher loss, the system creates a new model for this unique task. The model knowledge library is where the system keeps its growing collection of representative models whenever they are created. The system keeps getting better and more flexible as it faces more tasks. In FC$^3$, the model library works using a linear mapping function, while the feature library relies on the autoencoder neural networks. This new approach, FCL$^3$, has been tested and found to be better than other similar benchmarks in both classification and regression scenarios. Sun et al (2022) was the extension of Sun et al (2018). The major difference lay in the feature library, which was based on the simple linear mapping function in Sun et al (2018) instead of the autoencoder. That is why Sun et al (2022) is considered to have used Neural Network methodology while Sun et al (2018) did not.

Kozal and Wozniak (2023) introduced a neural network-based architecture that increases the depth of the network in a manner similar to a tree structure. The architecture is divided into three parts: a pre-trained model, referred to as the backbone, followed by intermediate layers that facilitate learning of current and future tasks, and finally, task-specific components that are unique to each task and retain knowledge about them. The key contribution of their approach is the adaptive shaping of the tree-like architecture when a new task is introduced based on its similarity to existing tasks. If two tasks are similar, new task-specific layers are attached to the intermediate layer, allowing knowledge sharing between the tasks. If tasks are dissimilar, the layers for the new task are attached directly to the backbone architecture. Similarity is measured by the entropy of predictions. During training, the backbone parameters are frozen, and only the shared and task-specific parameters are updated based on the ongoing tasks.

Other studies have also explored dynamic Parameter Isolation, including Pandit and Kudithipudi (2020), Ke et al (2021), and Gao et al (2022). In the context of Online Continual Learning, the Parameter Isolation remains valuable due to its adaptability and flexibility (Yoon et al 2017). So far, this study has mainly focused on studies that use neural networks in the Parameter Isolation category. However, not all studies rely on neural networks. In the following paragraphs, the authors will review studies that utilize Parameter Isolation techniques in non-neural network research.

Another study conducted by Ruvolo and Eaton (2013) introduces *“Active Task Selection for Lifelong Machine Learning”*, where a learner dynamically picks its next task to maximize future tasks performance, a.k.a, Curriculum Learning (Bengio et al 2009), diverging from traditional passive learning sequences. In this study, when a new task’s training dataset is introduced, a new regression-based model is quickly added to learn that specific task. However, when it comes to the optimization process for each model, only the model that corresponds to the ongoing task is updated. If a new task is introduced for the first time, a new regression model for that task is trained. Since the Task-IDs are known in their study, choosing the related model was not their concern. Ruvolo and Eaton (2013) evaluate two strategies: Information Maximization and Diversity Methods, against a random selection baseline. Integrated into the Efficient Lifelong Learning Algorithm (ELLA), Ruvolo and Eaton (2013)’s approach significantly boosts knowledge transfer, enhances the scalability of the lifelong learner, cuts down on learning time, and offers greater efficiency and adaptability.

In their work, Khan and Khalid (2017) leverages the storage of rules that pertain to both syntagmatic and paradigmatic relationships within topic modelling, an unsupervised learning problem. The concept of syntagmatic relationships is based on the positive and negative co-occurrence of words. A positive co-occurrence indicates a strong tendency for two words to appear together, while a negative co-occurrence suggests a low likelihood of the two words appearing together. On the other hand, paradigmatic relationships primarily focus on the similarity of meanings between words. As new topics are introduced within each task (e.g., a document), new rules are extracted, and they update the knowledge base to help future topic modelling. These rules can be considered as stored models that enable extracting the topics and learning new topics in a new task without requiring many samples, as previous knowledge is transferred through the rules. For instance, a rule can suggest that if two words (e.g., meal and eat) had positive syntagmatic in the past, this results in assigning a new document (task) to the same topic (e.g., food). These rules can help in assigning the documents to their related topics, even if they are short documents. Additionally, after assigning a document to a topic, the rules (relationship of words) are updated based on the new documents in the knowledge base to enhance KT. Notably, Khan and Khalid (2017)’s model outperforms both Automatic Must-link Cannot-Link (AMC) based topic models and Latent Dirichlet Allocation (LDA).

In their work, Benavides-Prado et al (2020) introduce Proficiente, a framework designed for knowledgeable supervised lifelong learning systems. Proficiente consists of two crucial components: *“AccGenSVM (Accumulative Generative Support Vector Machine) and HRSVM (Hypothesis Refinement Support Vector Machine).”* AccGenSVM is tailored for Forward Transfer Learning. It selectively transfers knowledge from a set of previous hypotheses, which are learned from past tasks, to a new task. These hypotheses represent the input-to-output mapping function of Support Vector Machines and are formulated based on the support vectors of each task. AccGenSVM utilizes Kullback–Leibler Divergence to identify the most suitable stored model for a recently presented task. On the other hand, HRSVM is employed for Backward Transfer Learning. It refines existing hypotheses by incorporating knowledge (support vectors) obtained from recent tasks, ensuring the retention of existing knowledge while integrating new information. The synergy between AccGenSVM and HRSVM within the Proficiente framework facilitates continuous learning and the gradual improvement of system performance over time. Each time, the hypothesis function of a task becomes another stored model, which is used for future task prediction.

In the approach proposed by Clingerman and Eaton (2017), a new Gaussian Process (GP) model is trained for each new task presented. This model is built on the knowledge transferred from previously trained models using a factorized formulation of the covariance functions, referring to a method for efficiently representing covariance kernels in GP models. In this regard, the negative log-marginal likelihood loss function is used to minimize and modify the covariance matrix based on overlapping previously learned tasks and new tasks to determine the coefficient (i.e., parameters or, in other words, covariance matrix arrays) of the new task while keeping the coefficient vectors related to previous tasks fixed. By minimizing this loss, covariance is shaped in a way that carries more knowledge about previous tasks and helps in the fast adaption to newly introduced tasks. GPs are non-parametric and provide a flexible method for regression, making them highly suited for lifelong learning scenarios where adaptability to new data is essential. The *“Gaussian Process Efficient Lifelong Learning Algorithm (GP-ELLA)”* transfers knowledge across tasks by modifying the covariance function of the GP. This modification allows the new model to leverage insights gained from previous tasks, resulting in improved learning efficiency and performance on new tasks. GP-ELLA has outperformed other multi-task GP methods and lifelong learning with linear models on various datasets, except for one synthetic dataset where ELLA, designed for linear models, performed slightly better.

#### Hybrid

A number of the researchers combined methods from two different KT techniques, and the authors classified these approaches under the Hybrid technique. This study identified three specific Hybrid combinations: “Replay & Regularization,” “Replay & Parameter Isolation,” and “Regularization & Parameter Isolation.”

***Replay & Regularization***: Gautam et al (2022) propose *“Task-free Generalized Continual Zero-shot Learning”* (Tf-GCZSL) and the problem formulation focuses on learning continually from a sequence of tasks, with the ability to handle unseen classes, which come with each new task, using a zero-shot learning framework. Knowledge Distillation (KD) and Replay methods are pivotal in this study. KD is a data-focused Regularization-based method and, in this study, helps the KT by benefiting from “dark knowledge”—once using an autoencoder, the latent spaces, which are information of encoded inputs, are known as dark knowledge. There are two memories: short-term memories for a current task and long-term memories for all previously seen tasks. Variational Autoencoders (VAEs) are used to encode the samples to latent space and store latent information along with the original samples in long-term memory. Once the samples of new tasks are directed to the long memory, VAEs store the information in a way that minimizes the loss of VAEs. Based on VAEs, four different losses (*“Kullback–Leibler divergence, reconstruction loss, Distribution-alignment loss, and Cross-alignment loss”*) are minimized with respect to different penalties for each loss. By doing so, the encoded samples in the buffer will not biased toward the recent task because of the penalties for the autoencoder’s parameter changes. The Replay method, particularly Experience Replay (ER) in this study, helps in reintroducing past stored samples and knowledge from the short-term and long-term memories to the model during training of new tasks. This combination of KD and ER enables the model to learn new tasks effectively.

The study by Gupta et al (2020) proposes a *“Lifelong Neural Topic Modeling”* (LNTM) framework that incorporates three strategies-*“Topic Regularization (TR) with TopicPool, Word-embedding Guided Topic Learning (EmbTF), and Selective-data Augmentation Learning (SAL)”*-to effectively manage the continual learning of topics. TR employs a Regularization method that penalizes the parameter updates that have leaned prior topics to not only transfer the knowledge about them when learning new topics but also not forget previous topics; meanwhile, in this step, maintaining a repository of past topics in the TopicPool occurs. Beyond the topics themselves, the EmbTF also incorporates pre-trained word embeddings accumulated in the WordPool to guide the learning process. This method pools the information contained in word embeddings and utilizes this accumulated multi-domain knowledge to help the topic modelling of future tasks. To complement these, the SAL technique expands the training dataset by selectively incorporating relevant historical documents as previous knowledge to be replayed in learning new topics. These methods together ensure that the LNTM framework not only mines relevant prior knowledge for new tasks but also retains this valuable information throughout the learning lifecycle. The accumulated topic and word embeddings in the TopicPool and WordPool act as a knowledge base, thus facilitating a robust and dynamic topic modelling process that adapts over time without losing the coherence of past learnings.

The study by Li et al (2023) introduces the *“Dynamic Consolidation Continual Learning”* (DCCL) method, an advancement in the domain of Regularization and Replay methods. DCCL distinguishes itself from the traditional Elastic Weights Consolidation approach with two key enhancements. First, it allows the model to explore the entire solution space rather than confining optimal weight choices to those nearby and heavily penalizing parameter changes. Second, important weights are dynamically penalized instead of receiving intense, fixed penalties. This ensures that parameters remain adaptable for future tasks, acknowledging that optimal values for these weights may evolve with the introduction of new tasks. Their method monitors changes in the loss function’s value, identifying specific parameters affecting loss function fluctuation more than parameters to impose greater penalties accordingly. Concurrently, to compute the loss function while also tracking it during the learning of a new task, access to data from previous tasks is essential. This requirement is fulfilled by the Replay technique, which utilizes a buffer to randomly store earlier samples and then replays these samples during the learning of a new task for loss function calculation. The combination of Replay and Regularization techniques in this study enhances the backward knowledge transfer effect as learning new tasks happens with respect to the important knowledge of previous tasks (i.e., parameters).

Boschini et al (2023) address KT by introducing eXtended-DER (X-DER), an updated version of Dark Experience Replay (DER). X-DER utilizes logit-based Replay, storing and replaying the logits,^4^ the samples, and their corresponding class labels of past examples. This includes "logits of the future past," which are updated logits that capture relationships with new classes encountered after the latest storage (classes observed after the last memory update and up to the current task at that time.). During training, when a past example is replayed, its stored logits are updated to include information about new classes, scaled appropriately to ensure they do not overshadow the ground truth logits. X-DER employs knowledge distillation by using a Regularization term that penalizes deviations from the stored logits, effectively constraining the model to retain its previous knowledge while learning new tasks. Additionally, a contrastive learning objective is integrated, preparing future classification by generating consistent representations through data augmentation. This involves creating multiple augmented versions of an example and encouraging the model to produce similar logits for these versions, thereby enhancing the model’s robustness to new classes. By replaying these enhanced logits and applying knowledge distillation, X-DER maintains high performance on both old and new tasks.

Tian et al (2023) propose a method that leverages KT based on latent feature similarity. The methodology involves three key steps: pre-trained and incremental models initialization, feature similarity transfer using a novel distillation loss based on centred kernel alignment (CKA), and Replay of old samples. Pre-trained models are used to initialize the network, which helps transfer knowledge and improve performance on new tasks instead of random initialization without knowledge. The feature similarity transfer is achieved through a CKA-based loss function that ensures the incremental model aligns its feature representations with those of the pre-trained model. Unlike the conventional L2 norm, which forces convergence of parameters and can limit generalization, CKA measures the similarity of neural network representations across different layers, exploiting the feature knowledge of the pre-trained model. Knowledge distillation is applied by encouraging the incremental model to mimic the pre-trained model’s output on stored samples, thus maintaining feature representation consistency. Dark Experience Replay, which uses logits of the last layer along with original samples and their labels, is integrated to store and Replay old class samples, allowing the network to prevent sudden distribution shifts.

***Replay & Parameter Isolation***: Han and Liu (2022) introduce the *“Selecting Related Knowledge via Efficient Channel Attention for Online Continual Learning (SRKOCL)”* framework, designed for enhancing knowledge transfer. This framework utilizes an efficient channel attention mechanism to identify the most pertinent knowledge for each specific task. In its architecture, attention blocks are strategically positioned following the convolutional layer. These blocks are instrumental in extracting the most relevant knowledge from both the samples stored in the buffer and the samples associated with new tasks. When a new task is introduced, these feature extractor models are re-trained using both the buffered samples and those from the new task. As such, these feature extractor models incorporate parameters pertinent to both new and previous tasks. During the learning of new tasks, the most relevant information is extracted through parameters activated by the channel attention mechanism, tailoring the learning process to the current task. This method ensures the automatic selection of the most important features for the current task. Meanwhile, their approach maintains a balanced buffer of stored samples as new tasks are introduced. In practical evaluations, their model exhibited comparable performance against various Regularization-based and Replay-based methods. It achieved heightened accuracy and more effective knowledge transfer on datasets like Split CIFAR-10, Split CIFAR-100, and SVHN.

Tessler et al (2017) propose *“Deep Hierarchical Approach to Lifelong Learning in Minecraft”* explores an innovative method for lifelong learning in the dynamic Minecraft environment, an open-ended game. It employs a Hierarchical Deep Reinforcement Learning Network (H-DRLN) integrated with Deep Skill Networks (DSNs) trained on specific subtasks. Deep Skill Networks are trained based on the sub-task and act like a pre-trained model that learns policies mapping functions (mapping states to optimal actions). In the training process of the DSNs, stored samples (i.e., states and associated actions) are used to Replay the previous actions (e.g., building, navigating, or crafting) in different states for transferring knowledge. After the availability of DSNs, H-DRLN decides if it will use pre-trained DSNs to determine the action or use its own mapping model to determine primitive action without DSNs. The paper showcases that this method, compared to standard Deep Q Networks, enhances performance and reduces the need for extensive data samples.

***Regularization & Parameter Isolation***: Cai et al (2022) introduce the *“Multimodal (i.e., visual and textual information) Structure-evolving Continual Graph Learning”* (MSCGL) model in dynamic graph learning. At its core, MSCGL excels in adaptively modifying its topological structure, which includes the arrangement of nodes and edges in the graph, as it incorporates new tasks. Each task is treated as a node classification problem in a multimodal graph. The model employs an *“Adaptive Multimodal Graph Neural Network”* (AdaMGNN) that expands both in architecture and parameters, accomplished by *“Neural Architecture Search (NAS) and Group Sparse Regularization (GSR).”* This combination ensures the model’s adaptability (i.e., KT) to new tasks through efficient architectures. The generation of new architectures through NAS is achieved using a recurrent network trained via reinforcement learning. This process maximizes expected architectural accuracy and enables the model to evolve with minimal human intervention. GSR, on the other hand, imposes block sparsity (group of parameters) on network parameters, which penalizes significant changes in the parameters related to previous tasks. These parameters are identified by a search method introduced in Pasunuru and Bansal (2019). The AdaMGNN, equipped with NAS and GSR, is considered a graph topology, where nodes indicate local computations and edges denote information connections. The MSCGL model’s effectiveness is demonstrated in real-world multimodal continual graph scenarios, showing robustness in adapting to varied task sequences and data distributions.

Chen et al (2023) address distribution adaptation using a Mixture-of-Experts (MoE) framework by progressively expanding the number of experts and gating dimensions. When new data distributions are introduced, they increase the number of experts without changing the network’s depth or width, thus avoiding an increase in computational costs. The gating mechanism, which assigns input tokens to the most suitable experts, is retrained to adapt to new distributions, activating only a subset of experts for each token to ensure efficiency. This sparse gating ensures that only the top-2 experts per token are activated during training and inference. To prevent overfitting and maintain knowledge from previous distributions, the authors use implicit Regularization via knowledge distillation, where outputs from old experts guide the training of new experts. Additionally, explicit Regularization is applied by freezing parameters of previously trained experts and gatings while only optimizing new ones. This combination of techniques allows the model to adapt to new data while preserving previously learned information, thereby enhancing knowledge transfer.

### Evaluation metrics and datasets

In this section, the focus is on evaluation metrics and benchmark datasets used in the selected studies. The section begins by discussing evaluation metrics employed in selected studies, highlighting their relevance and application in assessing KT effectiveness. It is followed by a discussion of the benchmark datasets. Although there is a broader body of study regarding lifelong learning metrics (New et al 2022), this study specifically concentrates on those pertinent to KT evaluation within LML.

#### Evaluation metrics

In Table 4, the first column lists the name of a metric used in the reviewed papers. The second column indicates the acronym, and the third column shows the frequency of use for each metric, representing how often it was employed across the selected papers. Finally, the third column provides a brief explanation of each metric, offering insights into what each metric measures. It is worth noting that Table 3 lists the associated papers that have used each of the listed metrics in Table 4.

**Table 4:** Metrics used in selected studies and their descriptions

Metric	Acronym	Frequency	Description
Average Accuracy	–	15	Measures the average accuracy across tasks, reflecting overall performance in multi-task scenarios
Forgetting Measure	–	6	Quantifies the degree to which a model forgets previously learned information
Root Mean Square Error	RMSE	4	Reflects the square root of the average squared differences between predicted and actual values
Reward	–	4	In reinforcement learning, indicates the reward achieved by an agent, measuring its performance
Area Under Curve	AUC	4	Measures the area under the ROC curve, indicating the ability to distinguish between classes
Backward Transfer	BWT	3	Assesses the impact of learning new tasks on the performance of previously learned tasks
Topic Coherence	–	2	Evaluates the coherence of topics in text mining, reflecting how meaningful the generated topics are
Time	–	2	Measures the time taken for a model to train or predict, indicating efficiency
Recall	–	2	Measures the proportion of actual positives that are correctly identified
Precision	–	2	Represents the proportion of positive identifications that were actually correct
Average Forgetting	FGT	2	Measures the amount of forgetting in continual learning scenarios
Learning Accuracy	LA	2	Measures the accuracy of a model while it is in the process of learning
F1-Score	–	2	The harmonic mean of precision and recall, providing a balance between the two
Perplexity	PPL	2	In language models, measures how well a probability model predicts a sample
Receiver Operating Characteristic Curve	ROC	1	A plot that illustrates the diagnostic ability of a binary classifier system
Cumulative Gain of Lifelong Learning	CGLL	1	Measures the cumulative improvement in learning across multiple tasks
Individual Accuracy	–	1	Measures the accuracy of individual tasks or classes in isolation
Normalized Accuracy	–	1	Represents accuracy normalized over different classes or tasks
Average Error	AER	1	The mean of all errors (difference between predicted and actual values) across predictions
Retained Accuracy	RA	1	Measures how well a model retains accuracy over time or across tasks
Average Displacement Error	ADE	1	Used in trajectory prediction, measures the average error in predicted vs actual positions
Final Displacement Error	FDE	1	Measures the error of the final predicted position against the actual final position
Mean Seen-class Accuracy	mSA	1	Average accuracy across classes that the model has seen during training
Mean Unseen-class Accuracy	mUA	1	Measures the model’s accuracy on classes not seen during training
Mean Harmonic Accuracy	mH	1	The harmonic mean of seen and unseen class accuracies
Number of Parameters	–	1	Counts the total number of trainable parameters in a model, indicating its size
Model Complexity	–	1	Evaluates the complexity of a model, which can affect performance and overfitting
Transfer Accuracy	–	1	Measures how well a model transfers knowledge from one task to another
Mutual Transfer	–	1	Assesses the bidirectional transfer efficiency between tasks in continual learning
Normalized Mutual Information	NMI	1	Measures the mutual information between two variables, normalized for comparison
Adjusted Rand Score	ARS	1	Evaluates the similarity of two assignments, ignoring permutations and with chance normalization
Homogeneity Score	HS	1	Assesses whether each cluster contains only members of a single class
Completeness Score	CS	1	Measures whether all members of a given class are assigned to the same cluster
V-measure Score	VM	1	The harmonic mean of homogeneity and completeness in clustering
Inlier Ratio	–	1	In computer vision, measures the ratio of inliers to outliers in feature matching
Registration Failure Rate	RFR	1	In image processing, measures the rate at which point cloud registration fails
Feature Matching Recall	FMR	1	Assesses the recall in the context of feature matching in images or point clouds
Forward Transfer	FWT	1	Evaluates the impact of previous learning on the performance in future tasks

In Table 4, commonly used metrics like average accuracy, RMSE, reward, and AUC are highlighted. However, the authors note that these metrics, while widely used, may not effectively represent KT as they are general performance indicators. To address this, the authors will detail formulas and descriptions for metrics they believe are more suited for evaluating KT. In what follows, the authors discuss in detail the metrics they believe are suitable for evaluating KT.

To assess models’ performance in terms of KT, the authors would recommend three traditional metrics from the LML literature: Average Accuracy, BWT, and FWT. These metrics are based on the study conducted by Lesort et al (2020), which is an improved version of the work originally presented by Lopez-Paz and Ranzato (2017).

Equation 1 shows average accuracy measure, where $R_{ij}$ is the test classification accuracy on task $t_j$ after observing the last sample from task $t_i$, $N$ is the total number of tasks. This average gives only a general picture of the performance of proposed KT techniques as it calculates the average on all seen tasks. Therefore, it is crucial to consider other metrics alongside average accuracy.

1 $$\begin{aligned} \text {Average Accuracy} = \frac{\sum _{i \ge j} R_{ij}}{\frac{N(N+1)}{2}} \end{aligned}$$

Equation 2 illustrates FWT, measuring the model’s capability to enhance its performance on new tasks using previous knowledge compared to starting from random status. Technically, the positive value of FWT denotes that earlier tasks could effectively help the model learn the new task. In contrast, negative values reflect the uselessness of previously acquired knowledge in learning new tasks. FWT is helpful as it assists in monitoring the forward knowledge transfer.

2 $$\begin{aligned} \text {FWT} = \frac{\sum _{i=1}^N \sum _{j=i+1}^N R_{ij}}{\frac{N(N-1)}{2}} \end{aligned}$$

Equation 3 defines the BWT, which assesses the impact of learning new tasks on the performance of previously learned tasks. This measure is crucial to ensure that acquiring new knowledge doesn’t negatively affect the performance on earlier tasks. Technically speaking, a positive value indicates the beneficial impact of a newly learned task on the understanding of previous tasks. Simply put, the knowledge gained from the recent task can enhance the model’s comprehension of earlier tasks. Conversely, a negative BWT signifies that the knowledge acquired from new tasks did not contribute positively to previous tasks, implying that the transferred knowledge was ineffective.

3 $$\begin{aligned} \text {BWT} = \frac{\sum _{i=2}^N \sum _{j=1}^{i-1} (R_{ij} - R_{jj})}{\frac{N(N-1)}{2}} \end{aligned}$$

Table 3 indicates that various studies, such as those by Benavides-Prado et al (2020); Gupta et al (2020); Yang et al (2022); Gao et al (2022); Gautam et al (2022); Mei et al (2022); Zhao et al (2022); Zaman et al (2023), have focused on developing new metrics or modifying existing ones for LML to facilitate the process of comparing their proposed approaches with benchmark models. Here, the authors present the relevant metrics that can assist in evaluating studies related to KT.

Benavides-Prado et al (2020) introduced a metric known as the CGLL, designed to evaluate the progressive improvement in a learner’s performance. It quantifies the amount of new information or knowledge accumulated over time. The formulation of the CGLL metric is demonstrated in Eq. 4. It assesses the accumulation of new knowledge by evaluating the extent of new information gained when learning a new task. This is done by comparing the performance of adapted models, specifically trained for the new task, in predicting the new task’s samples, against the performance of the previous model without any adaptations (i.e., before training with the new task’s samples). Here, the performance can be tracked by the changes in the accuracy value or other metrics.

4 $$\begin{aligned} CG(LL)_{t} = CG(LL)_{t-1} + \frac{1}{T_N} \sum _{i=1}^{T_N}P(y_{i}^{(s)}, f_{it}^{(s)})-P(y_{i}^{(s)}, f_{i(t-1)}^{(s)}) \end{aligned}$$

Where *CG* refers to the cumulative gain of leaner, and $CG(LL)_0$ is equal to zero as no knowledge has been gained at the start. *LL* presents Lifelong Learner, which can be any of the reviewed models. *t* denotes the timestamp, $y_i$ represents the *i*th samples ground-truth label, and $f^{(s)}$ shows the source mapping function (i.e., source hypothesis^5^). *P*(.) can be any metric, such as accuracy. *CG* grows based on the difference of *P*(.).

In their study on task-agnostic prediction, Gautam et al (2022) utilized three modified metrics to evaluate the continual Zero-Shot Learning (CZSL) approach. Their study is about class incremental, and all the classes of tasks seen so far $t-1$ are considered as *seen classes*, and all the classes of the future tasks ($t+1$ to *T*) are considered as *unseen* classes. To evaluate the model for the *t* th task in the context of task-agnostic prediction and how the seen classes knowledge can improve the accuracy of unseen classes in CZSL, the following evaluation metrics (Eqs. 5, 6, and 7) are employed:

5 $$\begin{aligned} mSA= & {} \frac{1}{T}\sum _{t=1}^{T}CAcc(D_{ts}^{\le t}, A^{\le t}) \end{aligned}$$

6 $$\begin{aligned} mUA= & {} \frac{1}{T-1}\sum _{t=1}^{T-1}CAcc(D_{ts}^{>t}, A^{>t}) \end{aligned}$$

7 $$\begin{aligned} mH= & {} \frac{1}{T-1}\sum _{t=1}^{T-1}H(D_{ts}^{\le t}, D_{ts}^{>t}, A) \end{aligned}$$

Where *mSA* refers to Mean Seen-class Accuracy and *CAcc* stands for each class accuracy, not the average of all class accuracies. *mUA* presents Mean Unseen-class Accuracy and *mH* denotes Mean Harmonic Accuracy (mH). *H* reflects the harmonic mean. In this context, $D^{\le t}$ represents all the training and testing samples from the first task up to the *t* th task, while $D^{>t}$ encompasses all the training and testing samples from the $(t + 1)$ th task to the final task in the sequence. *A* represents the actual labels. A higher value for the metrics indicates better model performance, and this well-performing model can effectively adapt to new tasks and classes within each task. The measures are beneficial as they assist in monitoring the model’s performance for each class in both seen and unseen scenarios. However, a potential drawback of this method is the possibility of neglecting to track overall performance. This issue becomes particularly pronounced in imbalanced scenarios, where class-specific accuracy might not provide a comprehensive view of the model’s performance, especially if it is biased toward the majority class.

In their work, Mei et al (2022) introduce a metric called Transfer Accuracy (TA), which is represented by Eq. 8. This metric is employed to assess the degree of relevance between different tasks. When an old task is relevant to a new task, one would anticipate that a well-performing model on the old task should also perform well on the new task. In simpler terms, the knowledge that has been acquired can be valuable for learning new tasks.

8 $$\begin{aligned} TA_{i,j}=\frac{1}{n_j}\sum _{j\epsilon n_j}{\mathbb {I}}(f(\theta _{j},x_j)\ne y_j) \end{aligned}$$

In this equation, *i* denotes the task number, and *j* signifies the model. The term $y_{j}$ is the ground-truth label for a sample $x_{j}$ from the test set of the *j*-th task, with $n_j$ representing the total number of testing examples for this task. The symbol ${\mathbb {I}}$ is used for the indicator function, while $\theta _{i}$ includes all the parameters of the trained model. The TA metric offers insight into the likelihood of achieving a well-performing model when the old and new tasks are correlated.

In the studies mentioned, which emphasize knowledge accumulation, zero-shot continual learning, incremental class learning, and the value of old knowledge, researchers have either created or adapted existing metrics to enhance their comparisons. Further details and analysis of these metrics, including the authors’ perspectives and usage patterns, will be discussed in Subsect. 6.2.

#### Benchmark dataset

Considering Table 3 and linking it to Table 2, one can notice that Supervised learning approaches predominantly use image datasets such as CIFAR and MNIST variations. This is likely due to the structured nature of these datasets, which are well-suited for tasks that require labelled data for classification. On the other hand, text and time-series datasets tend to be applied in unsupervised learning, where the goal is to discover patterns or groupings without pre-labelled outcomes. KT techniques like Parameter Isolation and Replay tend to be tested on diverse datasets, suggesting these techniques are being validated across different data modalities. Techniques that involve architectural modifications, such as dynamic architecture, are often paired with more complex datasets like ImageNet, which could benefit from such intricate model adjustments.

Table 3 shows that image-based datasets are preferred, which could be attributed to several reasons. Image data is inherently rich in complexity and diversity, making it a fertile ground for testing a wide range of KT approaches, from convolutional neural networks to dynamic graph-based methods. These datasets are not only plentiful but also come with established benchmarks, which makes them ideal for demonstrating the efficacy of KT techniques and comparing them against known baselines. The tables also show that while image data predominates, other types such as text and time series are also used, although to a lesser extent. Text data is likely chosen for its natural fit to NLP tasks, and time series data for problems requiring sequential analysis. Regarding stream data, its relative scarcity of use may be due to the complexities associated with processing real-time, continuously flowing data, which poses additional challenges that not all KT approaches may be equipped to handle.

The choice of datasets seems to be influenced by the domain of application, the complexity of tasks, the availability of benchmarks, and the data type’s alignment with the specific KT challenges addressed. For example, in computer vision applications, there are various datasets with different levels of complexity. These variations may include the number of classes in each task, the quality of images, and their diversified domain (e.g., object, numbers, animals) levels. As a result, researchers have more flexibility in evaluating their proposed approaches and thus prefer to focus on computer vision problems compared to others.

In this review, Appendix Table 6 serves as a comprehensive representation of the datasets used in the studies analyzed. It lists and categorizes datasets used among selected studies, giving a clear overview of the types of data used in various research efforts within this field. The *Dataset* column shows the name of each dataset, while the *Application* column indicates the area in which it was used. The *Dimension* and *Observations* columns provide information on the dimension of the dataset’s feature space and the number of samples in each dataset, respectively.

## Answers to the research questions

This section links the review findings to the research questions stated earlier.

### RQ1: What are the current and state-of-the-art techniques for KT in LML?

**Fig. 6:**
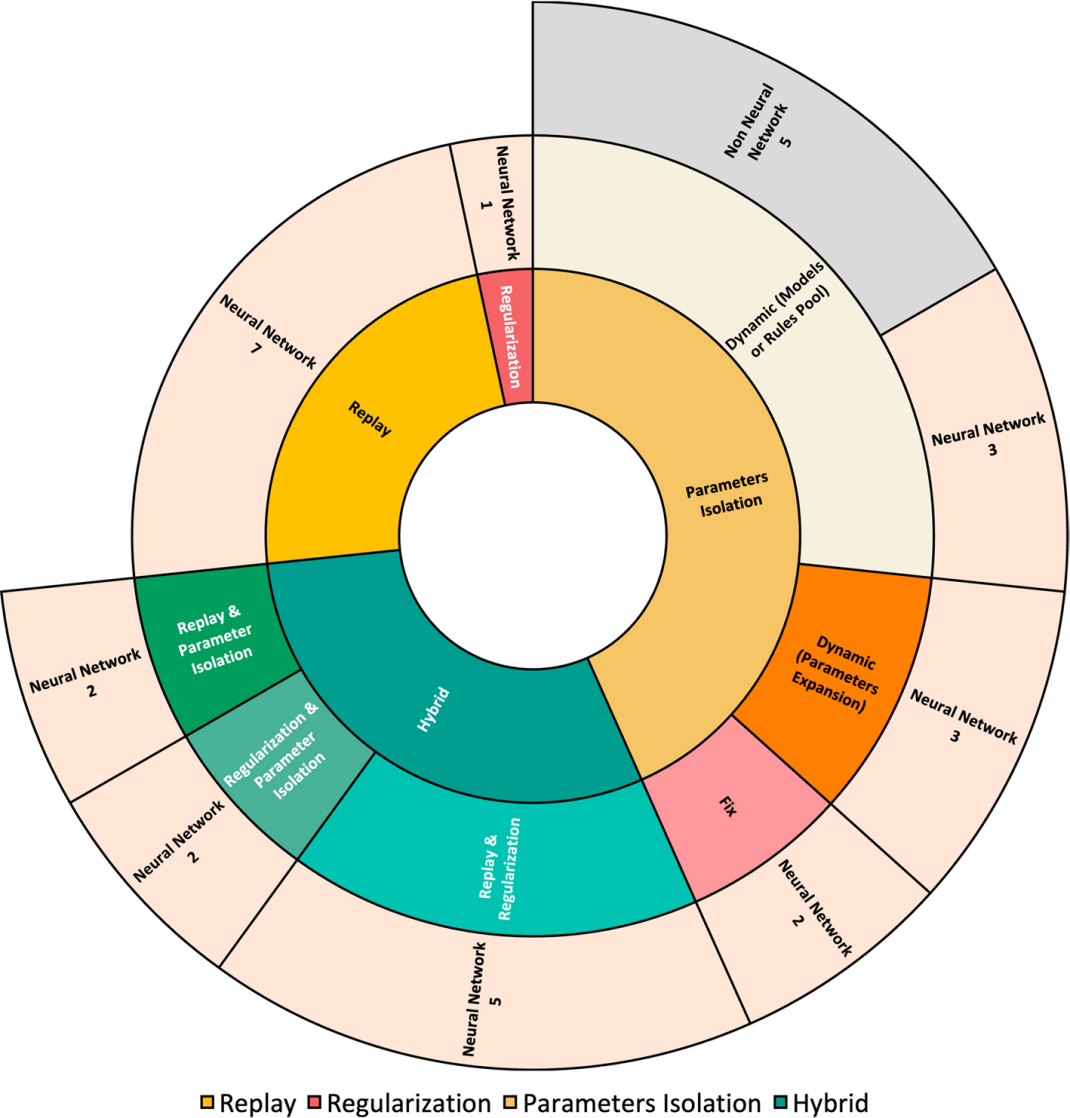
A summary of selected studies, categorized the related techniques used within each branch with respect to the methodology of being NN or non-NN

Drawing from Table 2 and insights from Sect. 5.1, additional graphs are introduced to illustrate the findings related to Research Question 1. Fig. 6 visually summarizes the KT techniques used in the selected studies, highlighting their distribution. Fig. 6 demonstrates that a significant portion of research implemented the Parameter Isolation Technique, owing to its notable benefits. Among these advantages is its versatility, which allows it to be integrated into various learning paradigms. Furthermore, unlike other techniques that have not delved into the potential of non-Neural Network modelling paradigms, models based on Parameter Isolation have initiated this exploration, thereby attracting additional attention to this approach.

Within the Parameter Isolation (Fig. 6), most of the studies implemented the Dynamic architecture over Fixed or Static one. Within the dynamic subset, the primary focus has been on storing or pooling models trained for each specific task, with the aim of transferring knowledge and refining each model to specialize in its respective task. As discussed in Sect. 5.1, these ’expert’ models interact with one another, sharing knowledge to enhance their collective understanding. For example, Sun et al (2022) and Sun et al (2018) utilized dual libraries for features and models. The feature library facilitated the identification of similarities between tasks and the sharing of relevant knowledge, while the model library maintained distinct models for each task as they were developed (for more detailed insights, refer to Sect. 5.1). The marked preference for Dynamic architecture within the area of Parameter Isolation merits attention, especially considering the method’s adaptability to non-neural network approaches.

Following Parameter Isolation, the Replay (=7 studies) and Hybrid techniques (=9 studies) were the most commonly employed methods. The authors argue that the Replay technique, with its notable advantages and compatibility with non-neural network models, presents significant opportunities for future research. They advocate this position because the Replay-based technique involves storing samples from previous tasks and revisiting them when learning new tasks. This process facilitates the transfer of useful knowledge and helps prevent the forgetting of earlier learned tasks. Consequently, in the IDL scenario, the Replay can be particularly effective as the samples maintain consistent input dimensions. However, in cases where the input samples’ dimensions vary across tasks, intermediary tools like autoencoders and encoded inputs can prove beneficial. Thus, when training a non-Neural Network model, these replayed samples can be reintroduced to the model, enhancing its efficiency by leveraging knowledge from past tasks. The authors contend that this potential has not been fully explored in the existing body of research.

A key observation from this study (Fig. 6) is the relatively infrequent use of Regularization techniques. This could be due to the inherent limitations of such methods, which include a fixed architecture and the challenge of identifying the appropriate penalty term. The static nature of the architecture in Regularization-based methods means that a higher penalty term results in concentration on previous tasks rather than transferring knowledge and adapting to new tasks through the parameters. Conversely, a lower penalty leads to a focus on more recent tasks, as preserving earlier knowledge becomes less critical when the penalty is smaller (Cai et al 2022). Furthermore, Regularization-based approaches become less effective as the number of tasks increases, leading to knowledge loss resulting from the calculation of derivatives and parameter updates in the long run (Mahmoud and Hajj 2022). Combining Regularization-based techniques with other methods has shown to be beneficial, especially when sample Replay or neural network parameter expansion is possible (refer to section 5.1.4). In such scenarios, Regularization techniques can provide options for Replay (=5 studies) or expansion of architecture (=2 studies) with respect to a penalty for significant changes. It is worth noting that Regularization-based techniques emerge as a more suitable option for privacy-related problems (Gunasekara et al 2022) as they do not keep original samples. This is why the unique advantages of using Regularization techniques on their own have not yet completely been overshadowed by the benefits derived from the combined approaches.

**Fig. 7:**
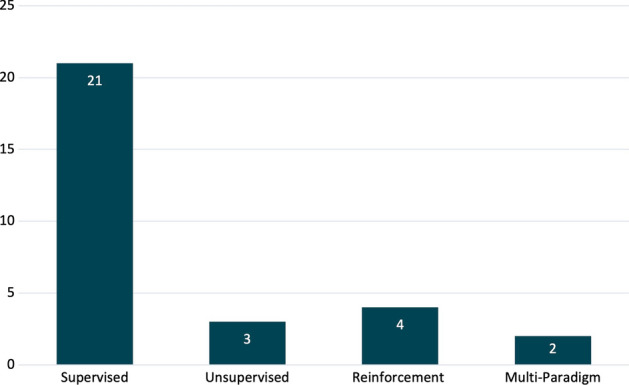
Distribution of selected publications based on used learning types

**Fig. 8:**
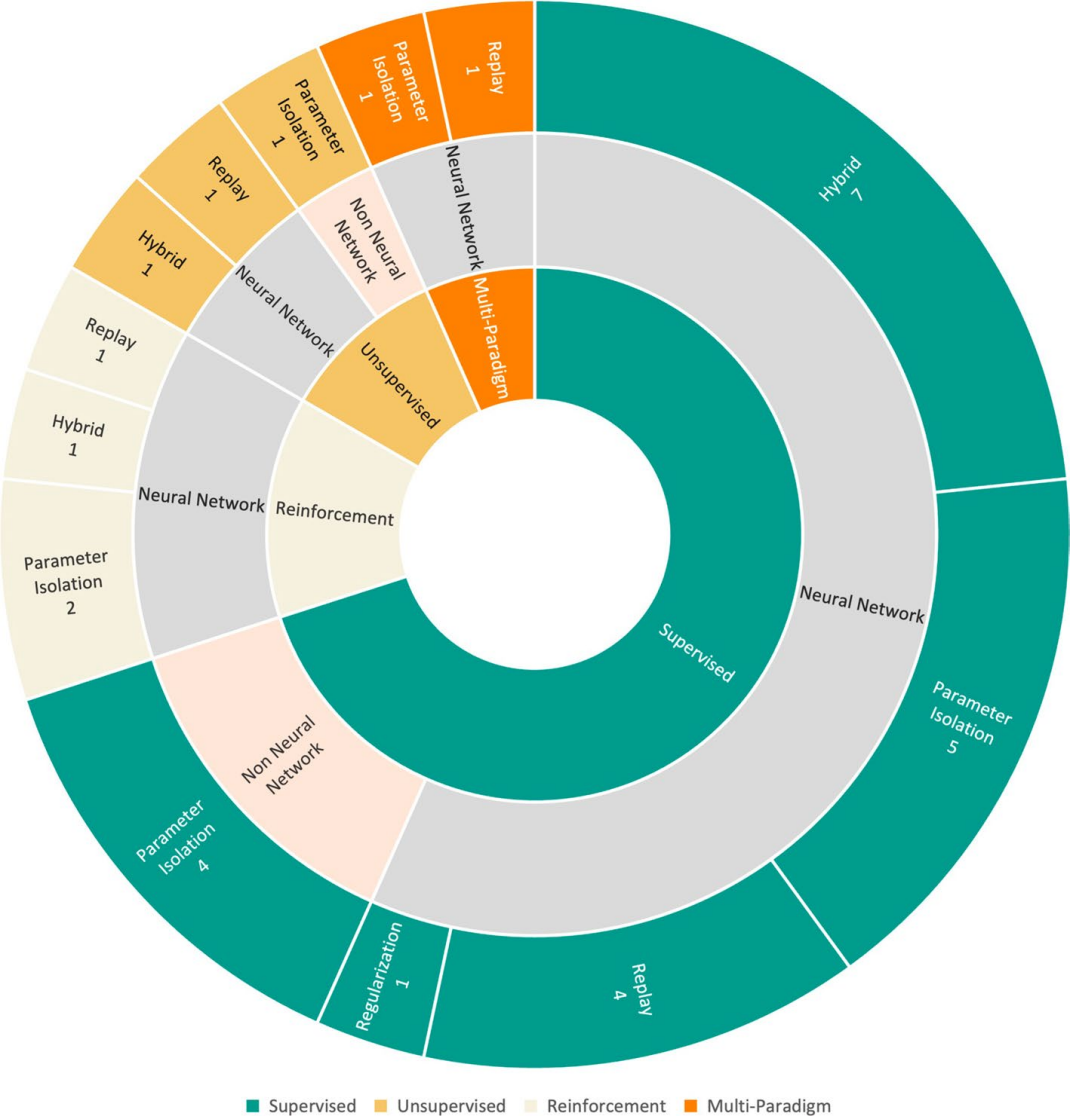
Portion of studies based on used learning types, knowledge transfer technique, and used methodology of NN or non-NN

Regarding the types of learning employed in the examined papers, Fig. 7 reveals that supervised learning is predominant, with 21 out of 30 papers discussing methods belonging to this class. This is a notable contrast to other learning paradigms, including unsupervised learning (3 papers), reinforcement learning (4 papers), and multi-paradigm approaches (2 papers). More details are shown in Fig. 8 with respect to learning type for non-NN and NN modelling paradigms. As shown, in supervised learning, Parameter Isolation (5 studies) and Hybrid (7 studies) techniques have a more substantial presence under the neural network sub-category. Under the supervised learning type with a non-NN modelling paradigm, one can notice that four studies are located under the Parameter Isolation group. Additionally, only one study based on an unsupervised learning paradigm has used non-NN, which is also based on the Parameter Isolation technique. Overall, five out of 30 studies have used non-NN modelling paradigms.

**Fig. 9:**
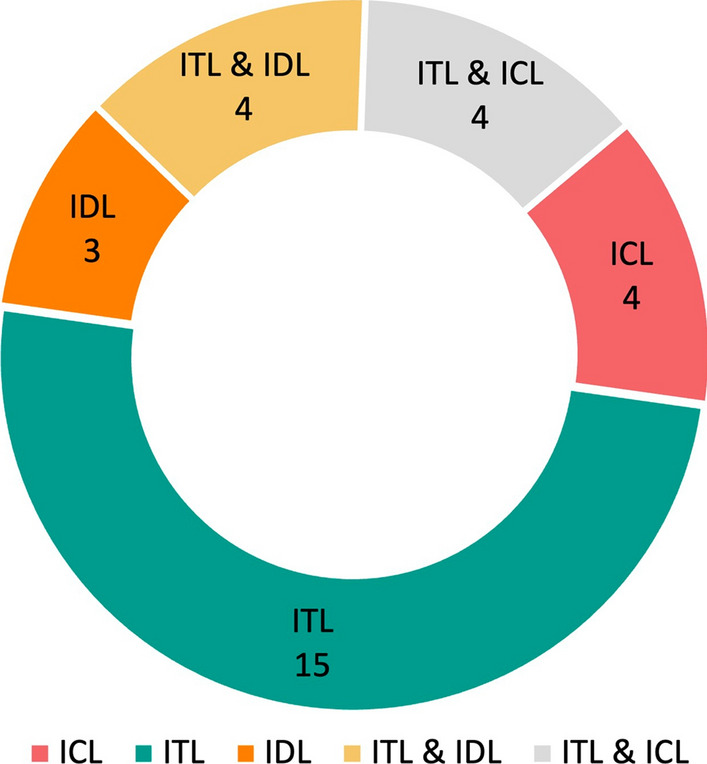
Portion of studies based on Incremental Domain Learning, Incremental Task Learning, and Incremental Class Learning

**Fig. 10:**
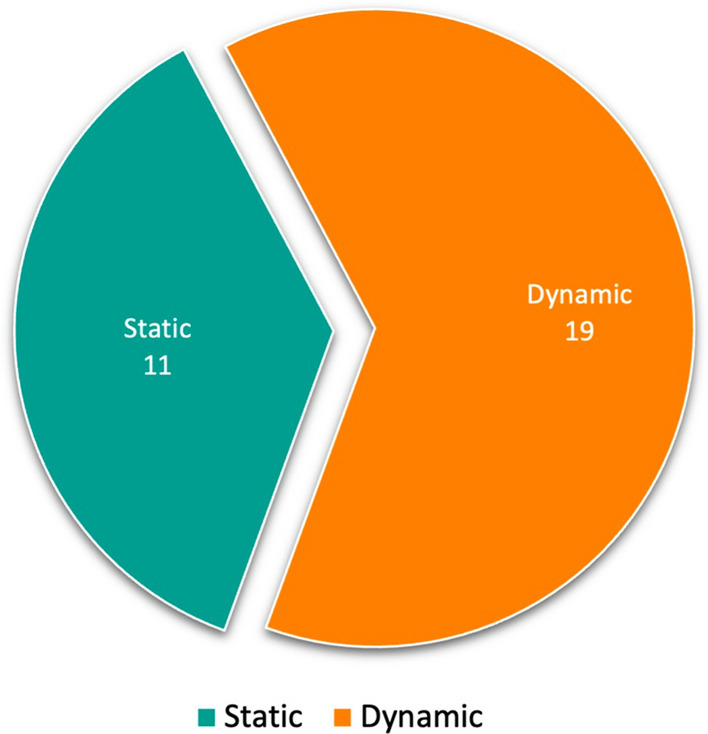
Employed Architecture based on the selected papers

Referring to Table 2 and the T/D/C and Architecture columns, the authors present Fig. 9 and Fig. 10 to elucidate their findings. Fig. 9 shows that most of the studies belong to Incremental Task Learning (= 15+4+4 studies in ITL), followed by Incremental Class Learning (=4+4 studies in ICL) and Incremental Domain Learning (=3+4 studies in IDL). This situation can be attributed to the availability of datasets and the flexibility that the ITL offers. Key advantages of ITL include the availability of end-of-task signals during training and testing phases. In contrast, Incremental Class Learning and Incremental Domain Learning present challenges such as increased computational demands and the need for end-of-task detectors.

Regarding the structure of the employed techniques, Fig. 10 provides further insights. It reveals that 19 out of the 30 papers favoured dynamic architecture. This can be attributed to the flexibility and enhanced performance offered by dynamic structures compared to static ones.

Table 2 shows that among the 30 papers reviewed, only 11 have made their code publicly accessible. This figure is expected to grow as the scientific community increasingly acknowledges the significance of transparency, replicability, and the validation of research findings. The importance of these factors is becoming more recognized, suggesting a trend towards open sharing of research materials and methods.

In terms of Statistical Testing, as per the S Test column in Table 2, it’s worth noticing that only two studies used statistical tests to validate the comparative performance of their models. Without rigorous statistical analysis, assertions about a model’s effectiveness remain tentative and necessitate further examination. This gap highlights a critical area for improvement in research methodology, emphasizing the need for more robust and statistically sound approaches to validate the claims made in scientific studies.

### RQ2: What evaluation metrics and benchmark datasets are utilized most frequently in studies focusing on KT in LML?

In this section, the authors will analyze the evaluation metrics, benchmark datasets, and the results provided by the selected studies with respect to the metrics and benchmark datasets.

#### Evaluation metrics

As stated earlier, eight of the 30 papers have introduced new or revised metrics, underscoring a growing emphasis on the development of specific evaluation metrics for LML. Among these eight studies, three concentrated on KT (see review in Sect. 5.2). Briefly, Benavides-Prado et al (2020) aimed to track the learner’s progression in assimilating new knowledge and to quantify the accumulation of newly acquired information. Consequently, they introduced the CGLL metric, as existing metrics were inadequate for this purpose. In the study by Gautam et al (2022) on Continual Zero-Shot Learning (CZSL), modifications to existing metrics were necessary as CZSL could not benefit from available ones, leading to the introduction of metrics like mSA, mUA, mH. In their research, Mei et al (2022) aimed to ascertain the extent of relevance between various tasks and to determine the degree to which previous tasks contribute to the comprehension of new tasks. Consequently, they introduced the TA metric as a means of quantifying this relationship.

**Fig. 11:**
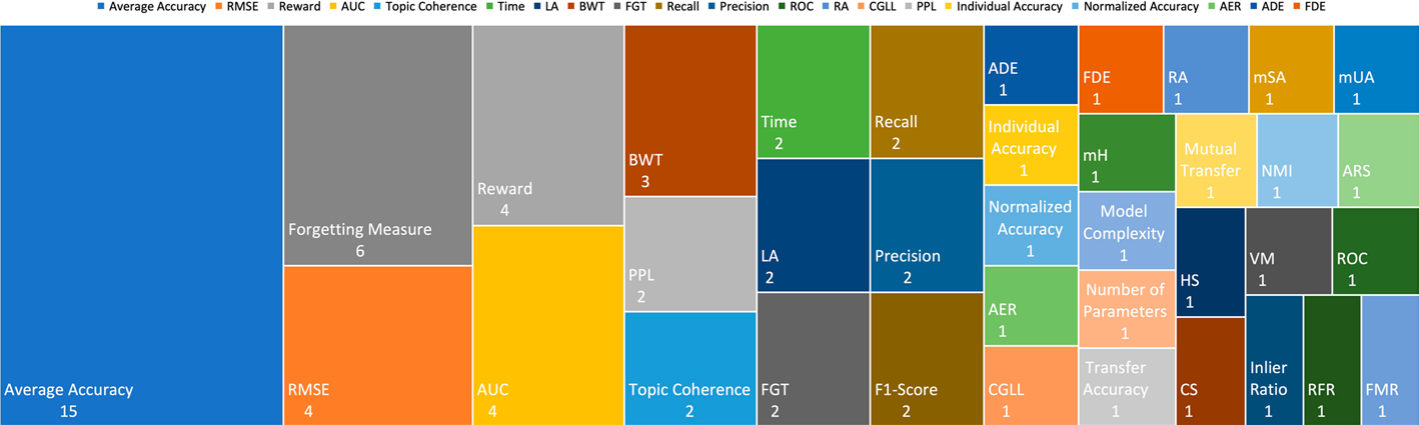
Frequency of used metrics for evaluating models in the selected papers, the tabular version of it was presented in Table 4

In LML environments, standard accuracy and related metrics often fall short in fully capturing the intricacies of learning across tasks. This is particularly evident when trying to understand how knowledge from one task affects another—a crucial aspect of LML and KT. To truly gauge the effectiveness of KT, we need metrics that can navigate complex scenarios, such as those with class imbalances in Incremental Class learning. The predominance of accuracy-centric metrics in existing studies points to a gap in the research. Although these metrics, including Average Accuracy, RMSE, Reward (Success Rate), and AUC, are frequently used (as depicted in Fig. 11), they are not specifically designed for LML contexts and often don’t address the unique challenges posed by KT in such settings. The absence of dedicated metrics for KT in LML may stem from the historical focus on single-task learning frameworks, where traditional metrics are sufficient. Moreover, the multifaceted nature of LML—where tasks can have a diverse range of classes and levels of balance-complicates the development of universal metrics. However, the evolving landscape of LML, with its emphasis on continuous adaptation and learning from a sequence of diverse tasks, underscores the necessity for specialized metrics. These would offer a more nuanced view of a model’s ability to retain and apply knowledge across various tasks, moving beyond mere accuracy to capture the depth and breadth of learning that takes place over time.

#### Benchmark dataset

Synthesis of the selected studies experiments across a group of contexts within LML reveals a dynamic exploration of the adaptability and efficiency of KT. Utilizing diverse benchmark datasets such as MNIST, CIFAR-100, ImageNet, CUB-200, CIFAR-10, and KITTI, the studies cover a wide array of domains, from image classification to human activity recognition; the results indicate that KT techniques are effective in the computer vision domain.

**Fig. 12:**
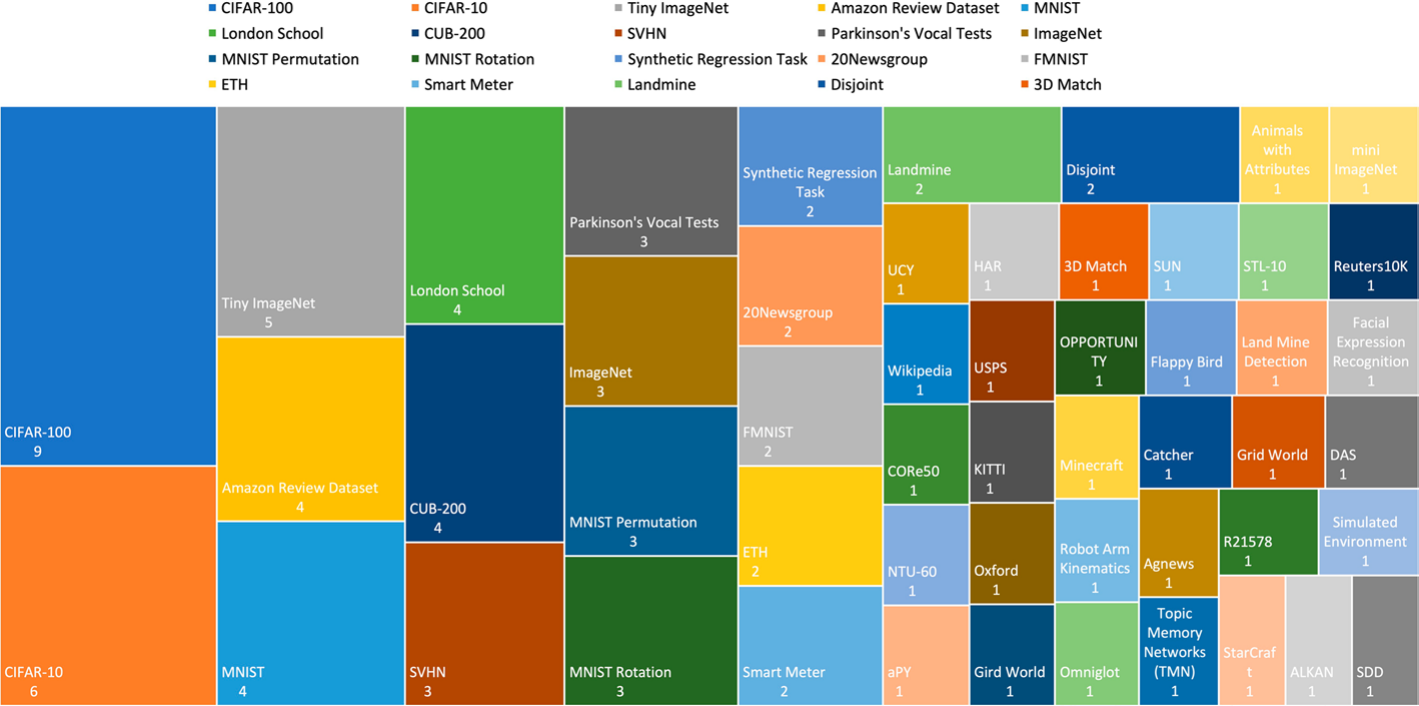
The treemap, derived from the data presented in Table 3, depicts the most commonly used datasets in selected papers regarding knowledge transfer techniques. The size of each rectangle corresponds to the number of papers utilizing the respective dataset

Figure 12 is a visual representation of datasets utilized in selected papers, with varying block sizes indicating the frequency of their usage across studies. Datasets such as “MNIST,” “ImageNet,” “CIFAR-100,” “CUB-200,” and “CIFAR10” are commonly used, reflecting their widespread adoption in the computer vision (CV) domain.

Figure 12 also shows that limited attention has been given to other areas, such as education with specialized datasets such as “London School” for educational data analysis, where LML is used for predicting exam scores of students in different schools, each defined as a task, “Land Mine Detection” for safety and engineering, specialized datasets for reinforcement learning and robotics such as “StarCraft” and “Robot Arm Kinetics.” Finally, datasets such as “Amazon Review Dataset” and “20Newsgroup” depict an application of KT in LML for text analysis. These observations suggest a growing recognition of LML’s potential across diverse fields, indicating an increased focus on areas outside CV.

#### Results provided by the selected papers

In their experimental analysis, Ruvolo and Eaton (2013) evaluated active task selection methods, including InfoMax, Diversity, and Diversity++ on datasets encompassing Land Mine, Facial Expression, Synthetic Data, and London School. The techniques were assessed for their efficiency in both general knowledge acquisition (overall learning from various tasks) and targeted knowledge acquisition (focused learning on specific tasks). Notably, selecting based on Diversity reduced the number of tasks required by up to 29.4% for general knowledge and 19.9% for targeted knowledge to outperform random selection, demonstrating its effectiveness in improving learning efficiency in LML systems by prioritizing more informative tasks. The research conducted by Tessler et al (2017) demonstrated that the H-DRLN architecture, which determines the usage of specific DSNs for mastering sub-tasks through skill reuse (actions tailored to the current state), achieved a remarkable success rate of 94 ± 4%, the highest among all neural networks assessed. This achievement was calculated from the mean success rates over the last ten epochs of the experiments, conducted within the Minecraft game across varied complexity levels (domains like navigation, item placement and picking up). The notable improvement in the H-DRLN’s success rate, facilitated by the reusing of skills (i.e, actions, a.k.a, knowledge) and further enhanced when integrated with the Double Deep Q-Network (DDQN), underscores its exceptional efficiency in handling the intricate challenges presented by Minecraft tasks.

Clingerman and Eaton (2017) showcases the GP-ELLA algorithm’s performance across various datasets, demonstrating significant improvements over other algorithms. For instance, on the “London Schools” dataset, GP-ELLA achieved a computation time of $489.8 \pm 18.7$ seconds, which is significantly lower than the Shared GP and MTGP methods, which required $13,070.5 \pm 1,784.9$ and $72,338.6 \pm 7,361.6$ seconds respectively. Similarly, for the “Robot Arm” dataset, GP-ELLA’s computation time was $38.3 \pm 0.2$ seconds, in contrast to $2,023.3 \pm 85.5$ seconds for Shared GP and $20,124.1 \pm 507.3$ seconds for MTGP, underscoring GP-ELLA’s efficiency and speed compared to the competing methods. Sun et al (2018) evaluated their proposed CL3 model and found that it surpassed the Multi-Task Learning (MTL) baseline in regression tasks, recording RMSE of 0.051, 0.024, and 0.157 on the Disjoint, Parkinson-Motor, and School Datasets, respectively. Additionally, the model demonstrated an improvement in AUC by 1.489% and 0.393% over the ELLA-Rand baseline for classification problems on the Landmine and SmartMeter datasets.

Riemer et al (2019) assessed the MER algorithm, leveraging the MNIST Permutations and MNIST Rotations benchmarks for evaluation. MER was juxtaposed with various baseline algorithms, including Online, Independent, Task Input, EWC, and Gradient Episodic Memory (GEM), with a relatively small memory buffer of 5,120 employed to facilitate learning across 20 tasks. The findings indicated that MER markedly surpassed these baselines in terms of RA, securing 89.56% on MNIST Rotations and 85.50% on MNIST Permutations. These results underscore MER’s enhanced capability in striking an optimal balance between KT and interference. Benavides-Prado et al (2020) evaluates selective KT methods, AccGenSVM and HRSVM, on synthetic and real-world datasets, demonstrating their superior performance in LML settings. AccGenSVM leverages privileged information from previous tasks to enhance learning efficiency, while HRSVM refines knowledge by transferring insights from new to previous tasks. AccGenSVM and HRSVM significantly outperform traditional SVMs, with HRSVM showing remarkable accuracy improvements of 24.4% on synthetic hyperplane, 12.7% on synthetic RBF, 3.7% on the 20 newsgroups, 2.6% on CIFAR-100, and 1.2% on ImageNet datasets, effectively utilizing prior knowledge to improve new task learning.

Pandit and Kudithipudi (2020) assessed the Relational Neurogenesis (RN) algorithm within two intricate Unity Engine-based simulations that mirror complex real-life situations. The first simulation involved a maze-navigating spider, while the second featured a rescue drone operating in a forest fire scenario, evaluating RN’s adaptability and retention capabilities. Quantitatively, RN outperformed existing algorithms like EWC, CHILD, and PathNET, demonstrating better cumulative scores. Specifically, in the spider simulation (Set A), RN achieved scores of 421, 470, 422, and 455 in the various sub-environments. In the drone simulation (Set B), scores were 71, 78, 66, and 81, respectively. These outcomes underscore RN’s advanced learning proficiency and illustrate its effectiveness in continuous learning and transferring knowledge. Ke et al (2021) conducted a well-organized evaluation phase, employing a statistical paired t-test to demonstrate the superiority of their proposed technique. Their proposed Knowledge and Accessibility Network (KAN) in continual sentiment classification, utilizing a dataset of Amazon reviews from 24 different product categories, treated as separate tasks. Each task comprised 2,500 positive and 2,500 negative reviews, with an 8:1:1 split for training, testing, and validation. KAN exhibited an impressive average accuracy of 85.24% across tasks and 87.99% on the final tasks, highlighting its effective knowledge transfer. In comparison, the ONE model achieved an average accuracy of 78.46% and 78.09% on the final tasks, while LSC recorded 82.19% average and 82.46% on the last tasks. These findings underscore KAN’s superior performance in sentiment classification, leveraging shared knowledge to outperform other models in both overall and task-specific status.

Li et al (2021) explores the proposed SLER mechanism within StarCraft II and GridWorld environments. SLER exhibited strong performance, securing a win rate exceeding 90% in GridWorld I and reaching 85% in StarCraft II following sequential task training. These outcomes highlight SLER’s capacity to handle concurrent task learning, effectively enhancing KT. This underscores its advantage in continuous reinforcement learning settings over EWC. Korycki and Krawczyk (2021) assessed the RSB algorithm efficacy in both stationary and class-incremental learning scenarios subject to concept drift, utilizing five image benchmarks: MNIST, FASHION, SVHN, CIFAR10, and IMAGENET10. In stationary sequences, RSB showcased strong KT abilities, with normalized average accuracies ranging from 99.67% on MNIST to 94.05% on IMAGENET10. When faced with drifting sequences, RSB exhibited remarkable adaptability, sustaining high performance with normalized average accuracies up to 99.38% on MNIST. This performance underscores RSB’s proficiency in navigating concept drift and the demands of continual learning.

Mahmoud and Hajj (2022) assessed the LOMA approach, utilizing the HAR and OPPORTUNITY datasets for human activity recognition, effectively demonstrating its capacity to KT. LOMA recorded an average accuracy of 81.72% on previously encountered tasks within the HAR dataset and 82.11% on newly introduced tasks from the OPPORTUNITY dataset. This performance distinctly surpasses that of alternative approaches such as LwF, Fine-Tuning (FT), MTL, and Single-Task Learning (STL), thereby underscoring LOMA’s adeptness at simultaneously acquiring new tasks and retaining previously learned information in time-series contexts. Cai et al (2022) evaluated MSCGL model using the Amazon and Articles datasets, demonstrating marked performance improvements. On the Amazon dataset, MSCGL attained an Average Accuracy of 89.44%, which, although marginally below the Joint Training model’s 90.04%, was considerably better than baseline methods like FT (75.61%), LWF (78.78%), and EWC (86.38%). For the Articles dataset, MSCGL achieved an 86.70% Average Accuracy, slightly below the Joint Training method’s 91.93%, but still surpassing baseline performances such as FT (61.01%) and EWC (82.17%). These outcomes suggest that MSCGL adeptly harnesses multimodal data and continuous learning approaches to sustain commendable performance across various tasks and datasets, with only a slight performance drop when compared to the optimal Joint Training approach.

Upon further examination, the Learning Accuracy (LA) values for the SRKOCL framework, as reported in Han and Liu (2022), are confirmed to be $0.9616 \pm 0.0051$, highlighting the model’s effectiveness in swiftly assimilating new tasks based of previous knowledge. Moreover, the FGT metric values were determined to be as minimal as $0.0511 \pm 0.0256$ on the Split SVHN dataset, demonstrating the model’s resilience in preserving earlier acquired knowledge amidst the integration of new data. In Gautam et al (2022), the method utilizing Tf-GCZSL notably surpassed the other baseline models, with performance enhancements exceeding 5%, 6%, 2%, 9%, and 2% for the CUB, aPY, AWA1, AWA2, and SUN datasets, respectively, when measured by the harmonic mean (H). Furthermore, the Tf-GCZSLMst method closely mirrored the performance of the theoretical offline upper limit, with only marginal deficits of 8.55%, 9.28%, 4.38%, 1.49%, and 8.14% for the corresponding datasets, also evaluated using H. This underscores its proficiency in enhancing KT. Zhao et al (2022) assessed the DBULL algorithm using the MNIST dataset, focusing on clustering quality metrics like NMI and ARS. DBULL recorded an NMI of 85.72% (with a standard error of 1.02) and an ARS of 83.53% (with a standard error of 2.35) on the MNIST dataset, surpassing comparative models such as DEC, VaDE, VAE+DP, CURL-F, and CURL-D in clustering effectiveness.

Zaman et al (2023)’s evaluation of the CMDGAT network concentrated on its correspondence accuracy and point cloud registration efficiency across various datasets, including Oxford, 3DMatch, KITTI, and ETH. This analysis measured the model’s proficiency in precisely matching and registering point clouds from diverse environments, showcasing its capabilities in handling point cloud registration tasks more effectively than existing approaches. CMDGAT exhibited a RFR of 18.45% and an IR of 33.17%, indicating its enhanced performance in comparison to competing methods. In Li et al (2023), the experimental analysis of the DCCL method focused on its performance on the Rotated MNIST and Permuted MNIST datasets. On Rotated MNIST, DCCL reached an Average Accuracy of 88.94% and a BWT of $-$0.09, showcasing substantial advancements in BWT when compared to methodologies like EWC, Memory Aware Synapses (MAS), and Synaptic Intelligence (SI). In the case of Permuted MNIST, DCCL achieved an ACC of 94.89% and a BWT of $-$0.02, surpassing these other approaches and underscoring its proficiency in continual learning endeavours.

Boschini et al (2023)’s dataset includes Split CIFAR-100, Split miniImageNet, and the newly introduced Split NTU-60. The proposed X-DER approach outperforms the other algorithms by achieving higher Average Accuracy (AA) and lower Forgetting Metrics (FM) metrics. On CIFAR-100, X-DER achieves an AA of 49.93% and FM of 19.90%, compared to DER++ which achieves an AA of 38.25% and FM of 50.54%. These improvements are attributed to X-DER’s strategies for memory updates and future preparation, which better preserve past knowledge and prepare for future tasks. Tian et al (2023) uses the Split ImageNet dataset, among others, to evaluate the proposed method PMKD, which shows better performance. PMKD achieved an AA of 71.85% and 71.92% on Split ImageNet-A and Split ImageNet-B, respectively, in incremental class learning scenarios, outperforming other methods such as DER++ (68.63% and 68.41%) and iCaRL (42.24% and 42.40%).

Kozal and Wozniak (2023) used datasets including Split CIFAR-100, Split Tiny ImageNet, and CORe50. The proposed approach outperformed other algorithms by achieving higher AA and lower FM metrics. On Split CIFAR-100, their method achieved an accuracy of 73% without pretraining and 80.91% with pretraining. On Split Tiny ImageNet, their method achieved an accuracy of 52.18% without pretraining and 58.11% with pretraining. For CORe50, their method achieved an accuracy of 99.55% (both with and without pretraining), outperforming ER which achieved 88.48% without pretraining and 89.5% with pretraining, with a FM of 0.0 indicating no forgetting. Using the earlier datasets and metrics, Chen et al (2023); Li et al (2024); Yu et al (2024) also demonstrated the efficiency of their proposed algorithms and approaches.

In summary, the experimental evaluations conducted by researchers in the field underscore the importance of task-specific adaptable architectures and algorithms, as seen in networks and methods such as CMDGAT and DCCL, which demonstrate significant improvements in specialized tasks. Furthermore, the effectiveness of KT mechanisms, as exhibited by the distilled H-DRLN and MER algorithm, is crucial for lifelong learning. The significant reduction in computation time by algorithms such as GP-ELLA emphasizes the role of computational efficiency, enabling quicker adaptation to new tasks. Incremental learning strategies, such as those evaluated through InfoMax and Diversity methods, enhance learning efficiency and resource management. The trade-off between generalizability and specialization remains a challenge, pointing to the need for tailored solutions in LML.

## Limitations

Despite the design and execution of the study following established systematic review principles, the study has the following limitations. Specifically, *exclusion of non-peer-reviewed papers* could lead to missing most recent publications that are in the process of peer evaluation. In the rapidly evolving domain of ML, many researchers choose to disseminate their findings as preprints, such as those found on ArXiv. Moreover, the exclusion of non-English language papers may result in missing work by some European and Asian researchers. Finally, despite using synonyms and wild cards in the query design, this study might miss papers within the LML paradigm that are published in venues not normally used by the ML research community. However, despite these limitations, this review provided valuable insights into the approaches to KT in LML.

To the best of authors’ knowledge, this study stands out in the LML reviews for three reasons: firstly, no *systematic literature review* has previously been conducted within LML, and secondly, it brings significant attention to the role of KT within LML, an area previously overlooked, and thirdly, past studies focused on NNs while this paper also looks into frameworks outside of NNs. Among the previous survey and literature review studies, Parisi et al (2019) have focused on addressing Catastrophic Forgetting (CF) within the context of non-stationary data distributions leading to CF. They examined methods devised to mitigate CF and briefly touched upon other learning paradigms inspired by LML and the human biological system, including Curriculum and Transfer Learning. De Lange et al (2022) undertook a survey and empirical analysis of the continual learning challenge, aiming to create a taxonomy and comprehensive review of cutting-edge models designed to deal with CF. Similar to Parisi et al (2019), the focus of De Lange et al (2022) was only on NNs studies. In another study, Lesort et al (2020) laid out definitions, strategies, and opportunities, with a particular focus on robotic applications. Their investigation centred around task-agnostic approaches, resource efficiency, and CF. In response to the research questions based on the mentioned aspects, their findings recommended using various metrics, concentrating on the MNIST and CIFAR datasets without predetermining the number of tasks beforehand, and considering constraints on available memory. Mai et al (2022) carried out an empirical investigation into online continual learning for image classification, demonstrating that the Maximally Interfered Retrieval (MIR), a Replay-based technique, surpassed other baseline methods across various domain and class incremental scenarios. Gunasekara et al (2023) carried out a survey on *"Online Streaming Continual Learning (OSCL),"* revealing that concept drift detection methods within the online learning paradigm could identify the onset of new tasks without relying on external signals. This finding paves the way for investigating task-agnostic capabilities within OSCL based on concept drift detection methodologies. Overall, the study at hand set itself aside from the other reviews by systematically focusing on KT in LML.

## Conclusion

To the authors’ knowledge, this systematic review of available techniques for KT in LML and the metrics and datasets used for evaluating them is the first comprehensive review on that topic. The study’s findings show that among KT techniques, Parameter Isolation methods are used the most, followed by Hybrid and Replay. This preference stems from advantages such as the exploration of the non-NN modelling paradigm and the flexibility that dynamic architecture in Parameter Isolation offers. Parameter Isolation allows for the development of methodologies that include maintaining a repository of models for expertly learning each task or the expansion of neural network architecture to more effectively learn new tasks. A substantial portion of the research has implemented KT techniques within the domain of supervised learning, with a particular emphasis on NNs, revealing potential for the application of KT methods across various learning paradigms. The authors suggest that the inherent adaptability of the Replay method positions it as a suitable option for incorporation into non-NN models, either as a standalone approach or in combination with Parameter Isolation, to enhance KT. This is feasible because it does not necessitate architectural changes, which might not be viable for non-NN models and could be effectively achieved through the manipulation of previously stored task samples. Another notable discovery is that, although Regularization-based techniques were less favoured, their integration with other methods in a Hybrid approach has gained considerable attention. It was also observed that more than half of the studies emphasized Incremental Task Learning as their developmental approach. Thus, the study also highlights the necessity of considering Incremental Domain and Class Learning in future research endeavours. In terms of validating the superiority of proposed techniques, only two out of the 30 studies relied on statistical tests. Consequently, this study encourages researchers to place greater emphasis on evaluating the significance of the superiority of their proposed methods. Furthermore, since only 11 papers have made their code publicly available, the authors also urge the LML community to share their code to promote replicability and transparency.

Regarding RQ2, it was found that while average accuracy is the most prevalent metric, there is a pressing need for metrics that can monitor cumulative knowledge, assess the relatedness of tasks, and extend to the area of continual zero-shot learning. In response, three studies introduced new and modified metrics to address these specific requirements. Although these proposed metrics represent a significant contribution towards overcoming the aforementioned limitations, they remain predominantly focused on accuracy. Consequently, future research should explore the potential of other metrics and customize them for LML in ongoing studies, including imbalanced matter in LML. The prevalent availability of public datasets for image-based applications, featuring a range of complexities and domains, has predominantly steered the focus towards the computer vision aspects of LML and KT. Hence, the introduction of datasets related to text and time series, with their inherent intricacies, could open new avenues for LML and KT applications within Natural Language Processing (NLP) and time series forecasting. In essence, while LML addresses the crucial challenge of sequentially learning multiple tasks and transferring knowledge between them, as opposed to learning a single task in traditional settings without leveraging such interactions, the area of NLP and time series applications has remained underexplored. The authors posit that a greater emphasis on studies utilizing unsupervised learning within LML and KT could help bridge this gap, given that these application areas often rely heavily on unsupervised learning problems. In contexts where data privacy is a concern, the literature has often recommended Regularization-based methods. However, the authors of this paper believe that the Replay method, particularly those based on pseudo-rehearsal approaches, along with Parameter Isolation, holds the potential to address privacy concerns. These methods could be increasingly relevant and find application in privacy-sensitive areas.

## Appendix: Search queries and benchmark datasets

Table 5 presents the applied query on each database. All the databases except ACM Digital Library follow the same search area; however, since ACM Digital Library cannot search different sections (Title, Abstract, and Keywords) simultaneously, the authors only benefit from the abstract section to complete the search. The dataset used in the selected papers is presented in more detail in Table 6.

**Table 5:** Query for each Database

Database	Query
Scopus	( TITLE-ABS-KEY ( “lifelong learning” OR “continual learning” OR “lifelong machine learning” ) AND TITLE-ABS-KEY ( experience OR ( knowledge PRE/3transfer* ) OR ( learning PRE/3 transfer* ) OR ( “transfer learning” ) OR information ) AND TITLE-ABS-KEY ( “fine-tuning” OR “regularization” OR “multi-task learning” OR “curriculum learning” OR “experience replay” OR technique* ) AND TITLE-ABS-KEY ( “machine learning” OR “deep learning” OR”artificial intelligence” OR “neural network” ) )
Web of Science	TS=(“lifelong learning” OR “continual learning” OR “lifelong machine learning”) AND TS=(experience OR knowledge near/3 transfer* OR learning near/3 transfer* OR “transfer learning” OR information) AND TS=(“fine-tuning” OR “regularization” OR “multi-task learning” OR “curriculum learning” OR “experience replay” OR technique*) AND TS=(“machine learning” OR “deep learning” OR “artificial intelligence” OR “neural network”)
IEEE Xplore	(“Document Title”:”Lifelong Learning” OR “Document Title”:”Continual Learning” OR “Document Title”:”lifelong machine learning” OR “Abstract”:”Lifelong Learning” OR “Abstract”:”Continual Learning” OR “Abstract”:lifelong machine learning OR”Index Terms”:”Lifelong Learning” OR “Index Terms”:”Continual Learning” OR “Index Terms”:lifelong machine learning) AND (“Document Title”: experience OR “Document Title”:knowledge near/3 transfer* OR “Document Title”:learning near/3 transfer* “Document Title”:”transfer learning” OR “Document Title”:information OR “Abstract”: experience OR “Abstract”:knowledge near/3 transfer* OR “Abstract”:learning near/3 transfer* OR “Abstract”:”transfer learning” OR “Abstract”:information OR “Index Terms”: experience OR “Index Terms”:knowledge near/3 transfer* OR “Index Terms”:learning near/3 transfer* OR “Index Terms”:”transfer learning” OR “Index Terms”:information) AND (“Document Title”:”fine-tuning” OR “Document Title”:”regularization” OR “Document Title”:”multi-task learning” OR “Document Title”:”curriculum learning” OR “Document Title”:”experience replay” OR “Document Title”:technique OR “Abstract”:”fine-tuning” OR “Abstract”:”regularization” OR “Abstract”:”multi-task learning” OR “Abstract”:”curriculum learning” OR “Abstract”:”experience replay” OR “Abstract”:technique OR “Index Terms”:”fine-tuning” OR “Index Terms”:”regularization” OR “Index Terms”:”multi-task learning” OR “Index Terms”:”curriculum learning” OR “Index Terms”:”experience replay” OR “Index Terms”:technique) AND (“Document Title”:”Machine Learning” OR “Document Title”: “Deep Learning” OR “Document Title”: “artificial intelligence” OR “Document Title”: “neural network” OR “Abstract”:”Machine Learning” OR “Abstract”:”Deep Learning” OR “Abstract”:”artificial intelligence” OR “Abstract”:”neural network” OR “Index Terms”:”Machine Learning” OR “Index Terms”:”Deep Learning” OR “Index Terms”:”artificial intelligence” OR “Index Terms”:”neural network”)
ACM Digital Library	Abstract:(“lifelong learning” OR “continual learning” OR “lifelong machine learning”) AND Abstract:(experience OR (knowledge PRE/3 transfer*) OR (learning PRE/3 transfer*) OR (“transfer learning”) OR information) AND Abstract:(“fine-tuning” OR “regularization” OR “multi-task learning” OR “curriculum learning” OR “experience replay” OR technique*) AND Abstract:(“machine learning” OR “deep learning” OR “artificial intelligence” OR “neural network”)

**Table 6:** Information of datasets employed for evaluating LML with respect to KT

No	Dataset	Application	Dimension	Observations	No	Dataset	Application	Dimension	Observations
1	London School	Exam Scores	–	15,362	27	Flappy Bird	Game AI development	–	–
2	Amazon Review Dataset	Sentiment analysis, text mining	Text data	Millions	28	Grid World	Reinforcement learning tasks	–	–
3	Parkinson’s Vocal Tests	Speech processing for medical diagnosis	Audio features	–	29	CIFAR100	Fine-grained object recognition	32x32 pixels	60,000 images
4	Caltech-UCSD Birds-200-2011 (CUB)	Bird species classification	312 binary attributes	11,788 images	30	Agnews	Text classification, news categorization	Text data	Millions
5	MNIST Permutation	Image recognition with permutation	28x28 pixels	70,000 images	31	Topic Memory Networks (TMN)	Text classification, topic modeling	Text data	–
6	MNIST Rotation	Image recognition with rotation	28x28 pixels	70,000 images	32	R21578 (Reuters-21578)	Text classification	–	10,369 documents
7	MNIST	Handwritten digit recognition	28x28 pixels	70,000 images	33	Simulated environment	Simulation for various ML tasks	–	–
8	SVHN	Street View House Numbers recognition	32x32 pixels	Over 600,000 digits	34	StarCraft	AI research in strategy games	Game environment data	-
9	Synthetic Regression Task	Regression tasks in machine learning	–	–	35	Gird World	Reinforcement learning tasks	Simulated environment	-
10	Disjoint	-	–	–	36	Fashion	Fashion image classification	28x28	70,000 images
11	Smart Meter	Energy consumption analysis	Time series data	–	37	CIFAR10	Object recognition in images	32 x 32 pixels	60,000 images
12	Landmine	Landmine detection	11	14,820	38	ALKAN	–	–	–
13	20Newsgroup	Text classification and clustering	Text data	20,000 documents	39	DAS	–	–	–
14	ImageNet	Image classification and object recognition	Varies	Over 14 million images	40	OPPORTUNITY	Human activity recognition	Sensor data	–
15	ETH	Pedestrian detection, tracking	Image/Video data	–	41	Human Activity Recognition (HAR)	Human activity recognition	Sensor data	–
16	Split CIFAR10	Image recognition tasks	32x32 pixels	60,000 images	42	Stanford Drone Dataset (SDD)	Autonomous drone navigation	Image/Video data	–
17	Split CIFAR100	Fine-grained object recognition	32x32 pixels	60,000 images	43	UCY	Crowd behavior analysis	Video data	–
18	Land Mine Detection	Landmine detection	Sensory data	–	44	Attribute Pascal and Yahoo (aPY)	Object recognition, attribute learning	Image data	–
19	Facial Expression Recognition	Facial expression analysis	Image/Video data	-	45	Animals with Attributes	Animal species classification	85	30,475 images
20	Minecraft	Game	–	–	46	Scene Categorization Benchmark (SUN)	Scene recognition and categorization	Image data	130,519 images
21	Robot Arm Kinematics	Robotics, motion analysis	–	–	47	CIFAR-100	Fine-grained object recognition	32x32 pixels	60,000 images
22	Split TinyImageNet	Image classification tasks	64x64 pixels	100,000 images	48	STL-10	Image recognition with fewer labels	96x96 pixels	100,000 images
23	United States Postal Service (USPS)	Handwritten digit recognition	16x16 pixels	9298 images	49	Reuters10K	Text categorization, clustering	Text data	10,000 documents
24	Many Permutation	–	–	–	50	Oxford	Visual object classes recognition	–	5062 images
25	Omniglot	Handwritten character recognition	105x105 pixels	1623 images	51	3D Match	3D scene matching and reconstruction	–	–
26	Catcher	–	–	–	52	KITTI	Autonomous driving tasks	–	–

## Footnotes

1 https://www.covidence.org/

2 https://github.com/

3 Soft Labels are like detailed clues from teacher-to-student models. They indicate not just the ground truth class but also the assigned probability for each class by teacher models, helping the student model to understand the nuance of decision-making.

4 The output of the penultimate layer before the softmax activation, representing the model’s confidence for each class.

5 The stored hypotheses (i.e., input-to-output mapping functions) refer to the source hypothesis, which can be adapted to be used in learning new tasks, in where it is referred to target hypothesis.
